# Toward 4D Biomaterials: Comparing Electrospun and 3D-Printed Shape-Memory Scaffolds

**DOI:** 10.3390/pharmaceutics18091084

**Published:** 2026-08-28

**Authors:** Luigi Ruccolo, Aleksandra Evangelista, Francesco Andresini, Rossella Dorati, Ida Genta, Marco Benazzo, Pietro Canzi, Elena Carlotto, Bice Conti, Silvia Pisani

**Affiliations:** 1 UOC Othorynolaringology, Fondazione IRCCS Policlinico San Matteo, Via Camillo Golgi 19, 27100 Pavia, Italy; l.ruccolo@smatteo.pv.it (L.R.); f.andresini@smatteo.pv.it (F.A.); marco.benazzo@unipv.it (M.B.); pietro.canzi@unipv.it (P.C.); elena.carlotto@unipv.it (E.C.); silvia.pisani@unipv.it (S.P.); 2 Department of Drug Sciences, University of Pavia, Via Torquato Taramelli 12, 27100 Pavia, Italy; aleksandra.evangelista01@universitadipavia.it (A.E.); rossella.dorati@unipv.it (R.D.); ida.genta@unipv.it (I.G.); 3 Integrated Unit of Experimental Surgery, Advanced Microsurgery, and Regenerative Medicine, Department of Clinical-Surgical, Diagnostic, and Pediatric Sciences, University of Pavia, Via Ferrata 9, 27100 Pavia, Italy

**Keywords:** shape-memory scaffolds, electrospinning, direct ink writing (DIW), dexam-ethasone

## Abstract

**Background/Objectives**: Shape-memory biodegradable scaffolds (4D scaffolds) represent promising platforms for minimally invasive tissue engineering and localized drug delivery. This study investigated how two different fabrication techniques, electrospinning (ES) and extrusion-based direct ink writing (DIW), influence the structural, thermal, mechanical, shape-memory, and drug-release properties of poly(L-lactide-co-caprolactone) (PLA/PCL 70:30) scaffolds loaded with dexamethasone (DXM). **Methods**: DXM-loaded PLA/PCL 70:30 scaffolds were fabricated by ES and DIW. The resulting matrices were characterized in terms of morphology, mass, thickness, drug-loading efficiency, thermal properties by differential scanning calorimetry, shape-memory performance, tensile mechanical properties, and in vitro DXM release. **Results**: Both fabrication techniques produced DXM-loaded matrices with comparable mass and thickness and high loading efficiencies (>82%). Glass transition temperatures ranged between 33 and 39 °C, supporting thermally induced shape recovery under physiologically relevant conditions, while ES processing was associated with higher polymer crystallinity. All scaffolds exhibited shape-memory behavior, with recovery ratios exceeding 90%. ES scaffolds displayed a microporous nanofibrous architecture, whereas DIW scaffolds showed a more open and highly porous structure. These morphological differences were reflected in their mechanical behavior: ES scaffolds exhibited higher tensile strength (up to 16.5 MPa vs. 1.9 MPa) and elongation at break (up to 320% vs. 243%). Drug-release profiles were also fabrication-dependent, with ES scaffolds reaching a plateau at approximately 80% DXM release, whereas DIW scaffolds showed near-complete release within 48 h. **Conclusions**: Both fabrication approaches preserved the thermoresponsive shape-memory behavior of PLA/PCL 70:30 but generated distinct scaffold architectures that strongly influenced mechanical performance and DXM-release kinetics.

## 1. Introduction

In recent decades, tissue engineering and regenerative medicine (TERM) have emerged as promising strategies to overcome the limitations of conventional transplantation, including donor shortages and immune rejection. By integrating biocompatible scaffolds, suitable cell sources, and biochemical cues, TERM aims to restore damaged tissues through the development of functional biomimetic constructs [1,2]. In parallel, incorporation of controlled drug delivery systems (DDSs) into polymeric scaffolds has transformed these products from passive structural supports into multifunctional therapeutic platforms capable of providing localized and sustained drug release while enhancing tissue regeneration [3,4].

Despite their therapeutic potential, clinical translation of these constructs remains limited by the need for invasive implantation procedures, which are associated with patient trauma, infection risk, and prolonged recovery [5]. Consequently, increasing attention has been directed toward minimally invasive implantation (MII), where deformable scaffolds can be delivered through small incisions or catheters and recover their original geometry in situ [6,7]. This concept has driven the development of Shape-Memory Polymers (SMPs), a class of smart materials capable of fixing a temporary shape and recovering their permanent configuration upon exposure to external stimuli, typically physiological temperature, moisture, pH, etc. [8,9]. By integrating MII with autonomous shape recovery, SMPs represent a powerful platform for the development of next-generation scaffolds capable of combining tissue regeneration with localized and controlled drug delivery.

Among shape-memory biomaterials, poly(lactic acid) (PLA) and poly(ε-caprolactone) (PCL) blends have attracted considerable interest owing to their biocompatibility, biodegradability, and tunable degradation kinetics [10]. Their shape-memory behavior depends on temperature changes and arises from the coexistence of permanent network points, provided by the crystalline or glassy PLA domains, and switching segments represented by the flexible PCL chains [11]. Heating above their $T_{g}$ enables temporary deformation, and subsequent cooling below $T_{g}$ fixes the polymer blend in the programmed shape. Subsequent exposure to physiological temperature conditions, which reside in a range higher than the polymer blend $T_{g}$, restores the polymer film or scaffold’s original architecture through the release of stored entropic energy [12]. PLA/PCL ratio can be adjusted to tailor degradation kinetics and synchronize scaffold resorption with tissue regeneration and sustained drug release, provided that it maintains suitable shape-memory behavior under physiologic conditions.

Electrospinning (ES) has long been regarded as one of the most effective techniques for fabricating PLA/PCL shape-memory scaffolds [13]. By producing nanofibrous meshes that closely resemble the extracellular matrix (ECM), ES provides scaffolds with high surface area and interconnected porosity, supporting cell attachment while enabling sustained drug release [14,15,16]. However, ES also presents significant limitations. The process mostly relies on organic solvents, is largely restricted to thin two-dimensional structures, and often generates densely packed fibrous networks with limited pore sizes that hinder deep cellular infiltration and vascularization [13,17].

To overcome these limitations, 4D printing has emerged as a promising manufacturing strategy by combining additive manufacturing with stimuli-responsive materials [18,19]. Unlike conventional 3D printing, 4D printing enables the fabrication of customized structures capable of undergoing controlled, stimulus-responsive transformations over time through programmed shape-memory behavior. Among the available additive manufacturing techniques, Direct Ink Writing (DIW) has attracted particular attention because it enables the extrusion of viscoelastic inks at room or mild temperatures, making it especially suitable for incorporating thermosensitive drugs and bioactive molecules without thermal degradation [20,21]. However, developing PLA/PCL-based DIW formulations that simultaneously provide suitable printability, structural stability, shape-memory behavior, and controlled drug release remains a significant challenge. Most current studies focus either on mechanically weak hydrogels or single-component polymers, highlighting the need for optimized drug-loaded PLA/PCL systems capable of bridging processability and functional performance [22].

Accordingly, this study aims to investigate electrospinning and DIW printing for the fabrication of PLA/PCL-based scaffolds, using the same polymeric formulation and incorporating dexamethasone (DXM) as a model drug to assess loading efficiency and drug release capability. The resulting constructs were systematically evaluated in terms of morphology, mechanical behavior, shape-memory properties, and drug delivery performance to elucidate how process-induced architectural differences influence their overall functionality. This comparative approach provides valuable insights for selecting optimal manufacturing strategies toward the development of next-generation biodegradable 4D scaffolds for minimally invasive tissue engineering and localized therapeutic delivery.

## 2. Materials and Methods

### 2.1. Materials

Methylene chloride (MC), 1,4-dioxane, and N, N-dimethylformamide (DMF), analytical grade, were purchased from Carlo Erba Reagents (Milan, Italy). Resomer^®^ LC 703 S, a poly(L-lactide-co-ε-caprolactone) (PLA/PCL) copolymer with a lactide-to-caprolactone ratio of 70:30 (mol/mol), a weight-average molecular weight ($M_{w}$) of 160,000 Da, and a $T_{g}$ of 32–42 °C, was supplied by EVONIK Operations GmbH (Darmstadt, Germany). Dexamethasone (DXM; $M_{w}=392.46$ g/mol), 4-(2-hydroxyethyl)-1-piperazineethanesulfonic acid (HEPES), and phosphate-buffered saline (PBS) were purchased from Sigma-Aldrich (Merck KGaA, Darmstadt, Germany). Dulbecco’s Modified Eagle Medium/Nutrient Mixture F-12 (DMEM/F-12) was obtained from Merck KGaA (Darmstadt, Germany). HPLC-grade acetonitrile was purchased from VWR International (Milan, Italy), while Milli-Q water was used throughout the study for chromatographic analyses and release experiments.

### 2.2. Electrospinning Manufacturing Technique

ES scaffolds were fabricated by electrospinning using a poly(L-lactide-*co*-ε-caprolactone) (PLA/PCL, 70:30 *w*/*w*) copolymer as starting material. The process was performed with a NANON 01 electrospinning apparatus (MECC Co., Ltd., Fukuoka, Japan) equipped with a controlled humidity system (MEEC Instruments, Pioltello, Italy), following the optimized and patented fabrication protocol previously reported by Pisani et al. [14]. Two polymeric solutions were prepared: one containing PLA/PCL (70:30) at 15% (*w*/*v*) dissolved in MC and DMF at a 70:30 (*v*/*v*) ratio, and one containing the same polymer concentration supplemented with 0.6% (*w*/*w*) DXM relative to the polymer content.

The electrospinning process was carried out under the following operating conditions: an applied voltage of 30 kV, a flow rate of 1 mL/h, a needle size of 22 G (0.7 mm), a syringe volume of 5 mL, a collector width of 40 mm at a speed of 10 mm/s, a cleaning frequency of 1 min, a collector-to-needle distance of 15 cm, and a total spinning time of 20 min.

ES placebo (EL-PBO) and DXM-loaded (EL-DXM) scaffolds were left for 12 h under a laminar flow hood to ensure complete solvent evaporation. The  obtained scaffolds were subsequently cut into rectangular specimens (1.5×3 cm) and characterized in terms of weight using an analytical balance (ENTRIS124-1S, Sartorius Lab Instruments GmbH & Co. KG, Göttingen, Germany) and thickness using a digital caliper (LTF, 0–150 mm).

### 2.3. Three-Dimensional Printing Manufacturing Technique

For the fabrication of DIW scaffolds, two polymeric solutions were prepared: one consisting of PLA/PCL (70:30) at 8% (*w*/*v*) dissolved in 1,4-dioxane, and another containing PLA/PCL (70:30) at 8% (*w*/*v*) supplemented with DXM at 0.6% (*w*/*w*) with respect to polymer. Both solutions were kept under magnetic stirring at room temperature until complete dissolution. This polymer concentration was selected following optimization of both ink formulation and printing parameters (reported in the Supplementary Materials S1.1) to obtain final DIW-printed constructs with weight and thickness comparable to those of dimensionally matched ES scaffolds, thereby ensuring a consistent baseline for subsequent comparative evaluations.

**Figure 1:**
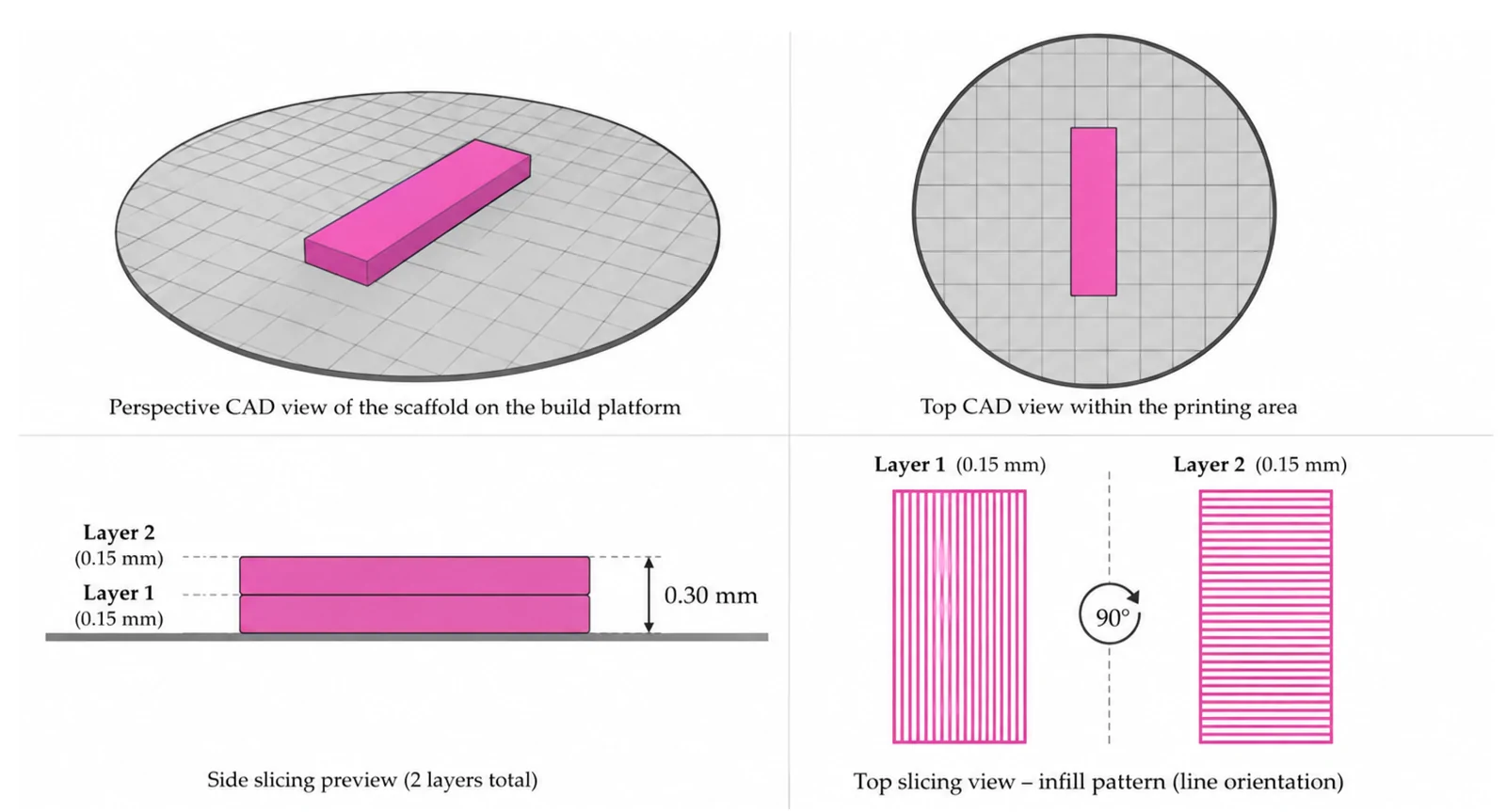
Computer-aided design (CAD) and slicing strategy used for the fabrication of DIW PLA/PCL scaffolds (1.5 × 3.0 cm). The workflow illustrates the scaffold design, layer-by-layer deposition approach, and orthogonal arrangement obtained by rotating the printing orientation between consecutive layers.

Three-dimensional scaffolds were fabricated by extrusion-based DIW using a Regemat 3D bioprinter (Regemat 3D, Granada, Spain) equipped with Regemat 3D software and operating through the Injection Volume Filling (IVF) system. The syringe-based extrusion printhead was equipped with a 5 mL syringe fitted with a 22 G Precision Tapered Dispensing Tip (polypropylene, inner orifice diameter = 0.016 in [0.406 mm]; Sigma-Aldrich, St. Louis, MO, USA). The effective deposition resolution used in this study was defined by the 0.406 mm tip inner diameter in the extrusion plane and by the programmed layer height of 0.15 mm in the vertical direction. A computer-aided design (CAD) model was developed to define the scaffold geometry and printing layout (Figure 1). The main printing parameters are summarized in Table 1.

The resulting polymeric solutions, indicated as DIW-PBO (placebo) and DIW-DXM (DXM-loaded), were deposited by the 3D printer onto Petri dishes, frozen at −80 °C, and  subsequently lyophilized using an Epsilon 2-4 LSCplus freeze dryer (Martin Christ Gefriertrocknungsanlagen GmbH, Osterode am Harz, Germany) operating at −50 °C and 0.2 mbar to ensure complete solvent sublimation. The obtained freeze-dried scaffolds were collected and characterized in terms of weight using the same analytical balance (ENTRIS124-1S, Sartorius Lab Instruments GmbH & Co. KG, Goettingen, Germany) and thickness using the same digital caliper (LTF, 0–150 mm) employed for the ES counterparts.

### 2.4. Thermal-Induced Shape-Memory Treatment (SMT)

The thermal SMT was carried out according to a customized programming and recovery protocol [16]. Both ES and DIW scaffolds were positioned between two metal plates (200 mm × 250 mm × 0.6 mm) and placed in an oven (Oven 52–80–120–240 L, Falc Instruments s.r.l., Treviglio (BG), Italy) at 60 °C for 30 min to ensure homogeneous thermal activation.

Subsequently, the samples were rolled around a stainless-steel mandrel with a diameter of 1.5 mm, following an adapted bending protocol to obtain a tube-shaped temporary configuration. After removal from the mandrel, the imposed deformation was fixed by freezing at −80 °C for 60 min ($R_{f,1}$ %).

The SMT scaffolds were subsequently dried under vacuum at room temperature for 24 h to remove residual water and to evaluate the stability of the temporary shape under dehydrated storage conditions (fixity ratio). Since water can act as a plasticizing agent in polymeric systems, its removal may affect chain mobility and potentially compromise shape fixation. Therefore, a second shape fixity ratio ($R_{f,2}\%$) was determined after vacuum drying to verify whether the temporary shape was maintained following dehydration prior to the shape recovery assessment.

To evaluate shape recovery, the dried scaffolds were immersed in PBS at 37 °C, a temperature within the $T_{g}$ range of the PLA/PCL copolymer. Recovery of the original geometry was quantified by calculating the shape recovery ratio ($R_{r}$ %).

The shape-memory performance was therefore assessed through three parameters: the shape fixity ratio after freezing ($R_{f,1}$ %), the shape fixity ratio after freezing and subsequent vacuum drying ($R_{f,2}$ %), and the shape recovery ratio ($R_{r}$ %), measured after immersing the scaffolds in PBS at 37 °C, calculated according to well-established mathematical models [23]:

(1) $$R_{f}\;\left(\%\right)=\left[1-\left(\frac{\varepsilon_{f}-\varepsilon_{i}}{\varepsilon_{f}}\right)\right]\times 100$$

where $\varepsilon_{f}$ is the deformation associated with the temporary shape, while $\varepsilon_{i}$ corresponds to the standard deformation imposed by the steel mandrel (diameter = 1.5 mm).

(2) $$R_{r}\;\left(\%\right)=\left(\frac{\varepsilon_{p}}{\varepsilon_{0}}\right)\times 100$$

where $\varepsilon_{p}$ represents the recovered strain and $\varepsilon_{0}$ is the initial strain corresponding to the original scaffold geometry.

The $R_{f,1}$, $R_{f,2}$, and $R_{r}$ values were determined through quantitative image analysis. Images acquired after freezing, after freeze-drying, and after recovery in PBS at 37 °C were processed using MATLAB R2025b (The MathWorks Inc., Natick, MA, USA) to obtain accurate dimensional measurements. The MATLAB script used for shape-memory image processing and quantitative analysis is provided in Supplementary Section S1.2. All analyses were performed in triplicate.

### 2.5. Dexamethasone Quantification

Each scaffold was dissolved in 1.0 mL of MC, followed by the addition of 2.0 mL of 0.1 M HEPES aqueous solution (pH 7.4) to induce phase separation. HEPES was selected as the aqueous phase to mimic physiological conditions and minimize analytical interference due to its low UV absorbance at 238 nm. Under these conditions, DXM exhibits preferential partitioning into the aqueous buffer phase due to its sufficient solubility in HEPES (∼70–75 μg/mL), while the PLA/PCL matrix remains in the organic phase, enabling effective liquid–liquid extraction [24]. The samples were then kept under agitation for 30 min to promote DXM transfer into the aqueous phase, followed by centrifugation at 6000 rpm for 20 min to achieve complete phase separation. Finally, the aqueous phase was carefully collected, filtered through a 0.22 μm membrane filter, and analyzed.

DXM quantification was performed using a 1260 Infinity HPLC system (Agilent Technologies, Santa Clara, CA, USA) equipped with a C8 Column Guard (4 × 30 mm, Phenomenex Ltd., Macclesfield, UK) and a ZORBAX C18 reverse-phase column (5 μm, 150 × 4.6 mm, Agilent Technologies, Santa Clara, CA, USA) [25]. The mobile phase consisted of Milli-Q water and acetonitrile (60:40 *v*/*v*) under isocratic conditions at a flow rate of 1.0 mL/min, with UV detection set at 238 nm. The calibration curve ($R^{2}=0.9985$, linear range 0.35–90 μg/mL) was constructed using DXM standard solutions prepared in 0.1 M HEPES buffer, which also served as the blank control.

The loading efficiency (LE%) was calculated according to Equation (3):

(3) $$\mathrm{LE}\left(\%\right)=\left(\frac{m_{\mathrm{DXM},\exp}}{m_{\mathrm{DXM},\mathrm{theor}}}\right)\times 100$$

where $m_{\mathrm{DXM},\exp}$ is the DXM amount experimentally quantified by HPLC and $m_{\mathrm{DXM},\mathrm{theor}}$ is the theoretical DXM content introduced during scaffold preparation. The extraction efficiency of the liquid–liquid extraction procedure was preliminarily evaluated by processing DXM standard solutions under the same experimental conditions, confirming quantitative drug recovery and the suitability of the method for subsequent HPLC analysis.

### 2.6. In Vitro Drug Release Test

In vitro drug release from the scaffolds was assessed in 0.1 M HEPES buffer at pH 7.4. Each scaffold was placed in an individual tube containing 5 mL of release medium [26]. The tubes were maintained at 37 °C under gentle magnetic stirring at 200 rpm throughout the entire release study.

At predetermined time intervals over 30 days, specifically 0.5, 1, 2, 3, 4, 5, and 6 h, and 7, 10, 21, 24, and 30 days, 1 mL aliquots were withdrawn from each tube and replaced with an equal volume of fresh HEPES buffer to maintain constant volume and sink conditions. Collected samples were filtered through a 0.22 μm membrane filter prior to analysis to remove suspended particles.

DXM concentration in each aliquot was quantified by HPLC using the same chromatographic conditions described in Section 2.5 and the previously established calibration curve. HEPES buffer was analyzed as a blank control to verify the absence of interfering chromatographic signals. The cumulative amount of released DXM was calculated from the measured concentrations and expressed as μg of DXM released per mg of scaffold and as a percentage of the initial drug content [27]. Release data were normalized according to the experimentally determined DXM extraction efficiency to account for recovery losses during the quantification procedure. All experiments were performed in triplicate, and results were reported as mean ± standard deviation.

To investigate the release mechanism of DXM from the scaffolds, the experimental release data were fitted to different mathematical kinetic models commonly used for controlled drug delivery systems. The cumulative percentage of drug released as a function of time was analyzed according to the zero-order, first-order, Higuchi, and Korsmeyer–Peppas models.

Model fitting was performed by linear regression using MATLAB R2025b (The MathWorks Inc., Natick, MA, USA), and the corresponding MATLAB code is provided in the Supplementary Materials S1.3.

The coefficient of determination ($R^{2}$) was used to evaluate the goodness of fit, and the model providing the highest $R^{2}$ value was considered the most representative of the release mechanism. For the Korsmeyer–Peppas model, only data corresponding to the initial 60% of cumulative drug release were considered, according to the assumptions of the model. The release exponent (*n*) was used to identify the dominant release mechanism, distinguishing between Fickian diffusion, anomalous transport, and polymer relaxation-controlled release [28].

### 2.7. Wettability

Wettability of ES and DIW scaffolds (PBO- and DXM-loaded) was evaluated by monitoring the apparent contact angle over time using the sessile-drop method. Measurements were performed using a DMe-211 Plus contact angle meter (Kyowa Interface Science Co., Ltd., Niiza, Saitama, Japan) equipped with a CCD (Charge-Coupled Device) camera and FAMAS software (Kyowa Interface Science Co., Ltd., Niiza, Saitama, Japan) [29]. Rectangular specimens (1.5×3.0 cm) were tested both before and after shape-memory programming cycles.

For each measurement, the scaffold was placed on the instrument plate and a droplet of testing medium, with a volume of 9±2μL, was deposited onto the scaffold surface. Measurements were performed at room temperature (25±2 °C). The FAMAS software automatically detected the droplet volume and geometrical parameters, including droplet height and base, and calculated the corresponding apparent contact angle at each acquisition time.

To investigate medium-dependent wettability, three aqueous media were tested for each scaffold formulation: HEPES buffer, phosphate-buffered saline (PBS), and Dulbecco’s Modified Eagle Medium (DMEM). HEPES buffer was selected as the release medium due to its ability to maintain a stable physiological pH during the release study. PBS was used as an isotonic saline medium mimicking physiological ionic strength, whereas DMEM was selected as a nutrient-rich cell culture medium containing inorganic salts, amino acids, glucose, and antibiotics [30,31]. The pH of all media ranged between 7.2 and 7.4.

For each droplet, contact angle values were recorded immediately after deposition and subsequently every 60 s up to 300 s, allowing the time-dependent wettability behavior of the scaffolds to be evaluated. Measurements were performed in triplicate on three independent scaffolds for each experimental condition.

### 2.8. Mechanical Test

The mechanical properties of PBO and DXM-loaded scaffolds, both before and after SMT, were evaluated by uniaxial tensile testing using a Mark-10 tensile tester equipped with a 25 N load cell (Mark-10 Corporation, Copiague, NY, USA).

Samples had a total length of 30 mm and a width of 15 mm. Specimen geometry was defined considering the dimensional scale of small specimens reported in Annex A of the ISO 527-2 standard [32], corresponding to the type 1BB specimens, which are characterized by a total length of at least 30 mm and a gauge length of 10 mm. Although the adopted specimens did not reproduce the dog-bone geometry prescribed by the standard, they maintained a comparable dimensional scale and an equivalent gauge region. During testing, failure was systematically verified to occur within the central gauge section.

Prior to mechanical testing, scaffold thickness was individually measured using an electronic digital caliper (LTF, 0–150 mm). For each specimen, thickness was calculated as the average of three independent measurements collected within the central gauge region and subsequently used for stress calculations.

Tensile tests were carried out at room temperature (25 °C) using a crosshead speed of 5 mm/min. Force and displacement were recorded during each test and subsequently processed using MATLAB software (R2025b, The MathWorks Inc., Natick, MA, USA) to generate engineering stress–strain curves. The MATLAB script used for tensile data processing is provided in the Supplementary Materials S1.5.

Engineering stress was calculated using the measured scaffold cross-sectional area, obtained from the specimen width and the average thickness determined for each sample. Engineering strain was calculated considering a gauge length of 10 mm. Young’s modulus was determined from the slope of the initial linear elastic region of the stress–strain curve. The maximum tensile stress ($\sigma_{\max}$) was defined as the highest stress reached during the test, while elongation at break ($\varepsilon_{b}$) was calculated from the strain corresponding to specimen failure and expressed as a percentage.

Force–displacement data were analyzed using a custom MATLAB routine. Briefly, the script automatically identified the effective starting point of each tensile curve using a moving-window approach requiring a monotonic increase in both force and displacement. This procedure allowed the exclusion of the initial portion of the acquisition potentially affected by instrumental noise, sample slack, or grip-settling artifacts.

After baseline correction, curves were truncated after the maximum load in order to analyze only the tensile response up to failure. Raw force and displacement values were then converted into engineering stress and engineering strain using the individually measured scaffold thicknesses, specimen width, and the defined gauge length.

The elastic region was identified by progressively fitting the initial portion of the stress–strain curve using the MATLAB polyfit function, applying a first-order polynomial to obtain the best linear approximation of the elastic region. The fitting interval was progressively extended until the coefficient of determination ($R^{2}$) remained above a predefined threshold.

For both ES and DIW scaffolds, the acceptance criterion was set at $R^{2}\geq 0.99$ with a minimum of 20 data points. These parameters were selected to ensure robust and reproducible identification of the linear elastic region despite the different structural organization of the two scaffold architectures [33].

For each scaffold, Young’s modulus, maximum tensile stress, and elongation at break were extracted from the stress–strain curves. Results were grouped according to scaffold type and treatment condition and expressed as mean ± standard deviation. All analyses were performed in triplicate.

### 2.9. Differential Scanning Calorimetry (DSC)

Differential scanning calorimetry (DSC) was employed to investigate the thermal behavior of the pristine polymer and scaffold formulations before and after SMT and to evaluate transition temperatures and associated enthalpy changes according to international standards [34].

DSC analyses were performed on pristine PLA/PCL 70:30 copolymer powder and on all scaffold formulations, including ES and DIW PBO and DXM-loaded scaffolds, both before and after SMT.

Thermal analyses were carried out using a Q2000 differential scanning calorimeter interfaced with a TA 5000 data station (TA Instruments, New Castle, DE, USA). The instrument was calibrated using an ultrapure indium standard with a purity of 99.999%, a melting temperature of 156.6 °C, and an enthalpy of fusion of $28.54\;J\cdot g^{-1}$.

Approximately 10–12 mg of each sample was weighed into standard open aluminum pans and analyzed under a nitrogen atmosphere with a purge flow of $45\;\mathrm{mL}\cdot \min^{-1}$. Samples were scanned from 0 to 200 °C at a constant heating rate of 10 °C·min ^−1^.

Thermographic traces were analyzed, and $T_{g}$, crystallization temperature ($T_{c}$), melting temperature ($T_{m}$), and the corresponding transition enthalpies (ΔH) evaluated.

### 2.10. Scanning Electron Microscopy (SEM)

The morphology of ES and DIW scaffolds, before and after SMT, was investigated by SEM.

Scaffold specimens were cut into square sections measuring 0.5×0.5 cm and mounted onto adhesive carbon tabs fixed on aluminum stubs. Prior to imaging, samples were coated with a conductive carbon layer using a Cressington 208 Carbon High Vacuum carbon coater (Cressington Scientific Instruments Ltd., Watford, UK) in order to minimize charging effects during electron beam exposure. The deposited conductive coating had an estimated thickness of approximately 10–15 nm.

SEM analyses were performed using a TESCAN MIRA3 XMU field-emission scanning electron microscope. Images were acquired under high vacuum conditions using an accelerating voltage of 5 kV, a working distance of 15 mm, and a secondary electron detector.

Both PBO- and DXM-loaded scaffolds, fabricated by ES and DIW, were analyzed before and after SMT. All analyses were performed in triplicate.

SEM micrographs were subsequently processed using MATLAB software (R2025b, The MathWorks Inc., Natick, MA, USA) for quantitative morphological analysis. The MATLAB script used for image processing and morphometric quantification is provided in the Supplementary Materials S1.6. For ES scaffolds, analysis included fiber diameter distribution, pore size, and apparent porosity, whereas for DIW scaffolds, pore size and porosity metrics were evaluated.

Briefly, SEM images were converted into binary masks through grayscale thresholding to distinguish solid polymer regions from void areas. Morphological parameters were then extracted from the segmented images using custom MATLAB routines. Fiber diameter measurements in ES scaffolds were obtained from multiple fibers randomly selected across different image regions, while pore size was calculated from the equivalent diameter of the segmented pore areas. Apparent porosity was determined as the ratio between the void area and the total image area and expressed as a percentage (%) [35].

This image-processing workflow enabled a quantitative comparison of scaffold architecture and the evaluation of the effects of DXM loading and SMT on scaffold morphology.

### 2.11. Statistical Analysis

All experiments were performed in triplicate unless otherwise specified, and the data are presented as mean ± standard deviation (SD). Statistical analyses were performed using MATLAB R2025b (The MathWorks Inc., Natick, MA, USA). All MATLAB codes used for data processing and statistical analysis are provided in the Supplementary Materials.

For shape-memory characterization, statistical comparisons between PBO and DXM-loaded scaffolds were performed using an unpaired two-tailed Student’s *t*-test. Differences were considered statistically significant when p<0.05.

For drug release kinetic analysis, experimental release data were fitted by linear regression to zero-order, first-order, Higuchi, and Korsmeyer–Peppas models. The goodness of fit was evaluated using the coefficient of determination ($R^{2}$), and the model with the highest $R^{2}$ value was considered the most representative of the release mechanism.

## 3. Results

### 3.1. Scaffold Fabrication and Dimensional Characterization

Scaffolds were successfully fabricated by electrospinning and DIW as rectangular specimens measuring 1.5 × 3.0 cm. Weight and thickness comparison of the PBO and DXM-loaded formulations showed that both fabrication techniques produced scaffolds with comparable weights, in the rank 11.94 ± 0.60 mg for ES PBO and 11.54 ± 0.14 mg for DIW PBO, as shown in Table 2. Thickness values remained within the sub-millimetric range for all samples, measuring 0.06 ± 0.01 mm for both ES formulations and approximately 0.10 ± 0.02 mm for DIW scaffolds. Overall, the dimensional characterization confirmed that the optimized fabrication conditions yielded constructs with comparable mass and closely related thickness values, providing a consistent basis for subsequent comparative analyses.

### 3.2. Shape-Memory Performance

**Figure 2:**
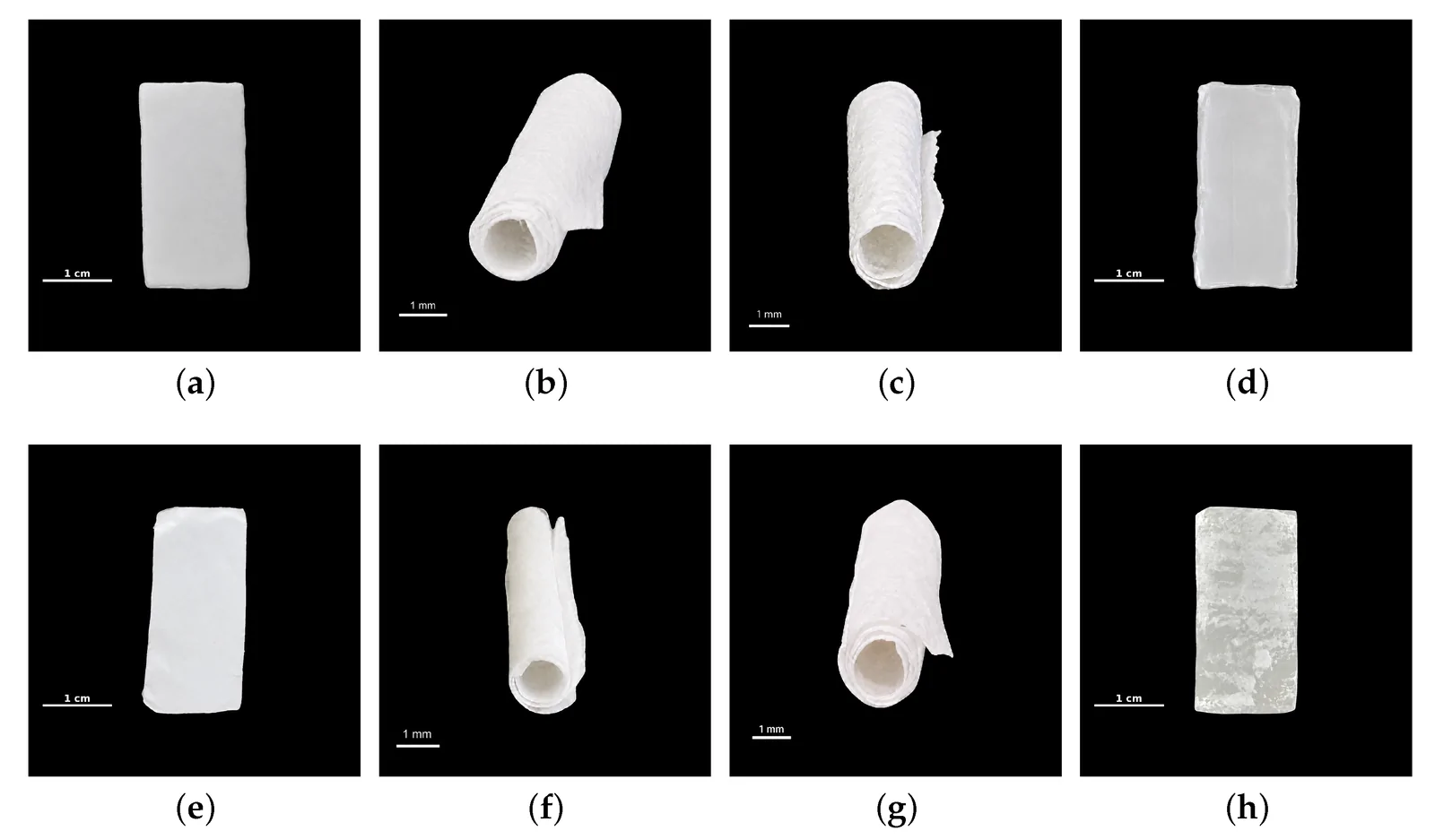
Representative macroscopic images of the shape-memory programming–fixation–recovery sequence of PBO PLA/PCL scaffolds fabricated by DIW and ES. DIW scaffolds are shown in panels (**a**–**d**), corresponding to the initial permanent configuration before SMT (**a**), the temporary configuration after fixation at −80 °C used for $R_{f1}$ evaluation (**b**), the temporary configuration retained after vacuum drying used for $R_{f2}$ evaluation (**c**), and the recovered configuration after immersion in PBS at 37 °C used for $R_{r}$ evaluation (**d**). ES scaffolds are shown in panels (**e**–**h**), following the same sequence: initial permanent configuration before SMT (**e**), the temporary configuration after fixation at −80 °C used for $R_{f1}$ evaluation (**f**), the temporary configuration retained after vacuum drying used for $R_{f2}$ evaluation (**g**), and the recovered configuration after immersion in PBS at 37 °C used for $R_{r}$ evaluation (**h**). Representative PBO scaffolds are shown for visualization of the programming sequence.

The shape-memory performance of ES and DIW scaffolds was evaluated by determining the shape fixity ratio after freezing at −80 °C ($R_{f1}$%) and after drying ($R_{f2}$%), together with the shape recovery ratio ($R_{r}$%). Representative macroscopic images of the investigated programming–fixation–recovery sequence are shown in Figure 2. For clarity, representative PBO scaffolds are reported for both fabrication techniques. The images illustrate the initial permanent configuration, the temporarily programmed shape after fixation at −80 °C, the configuration retained after vacuum drying, and the recovered shape after immersion in PBS at 37 °C.

**Figure 3:**
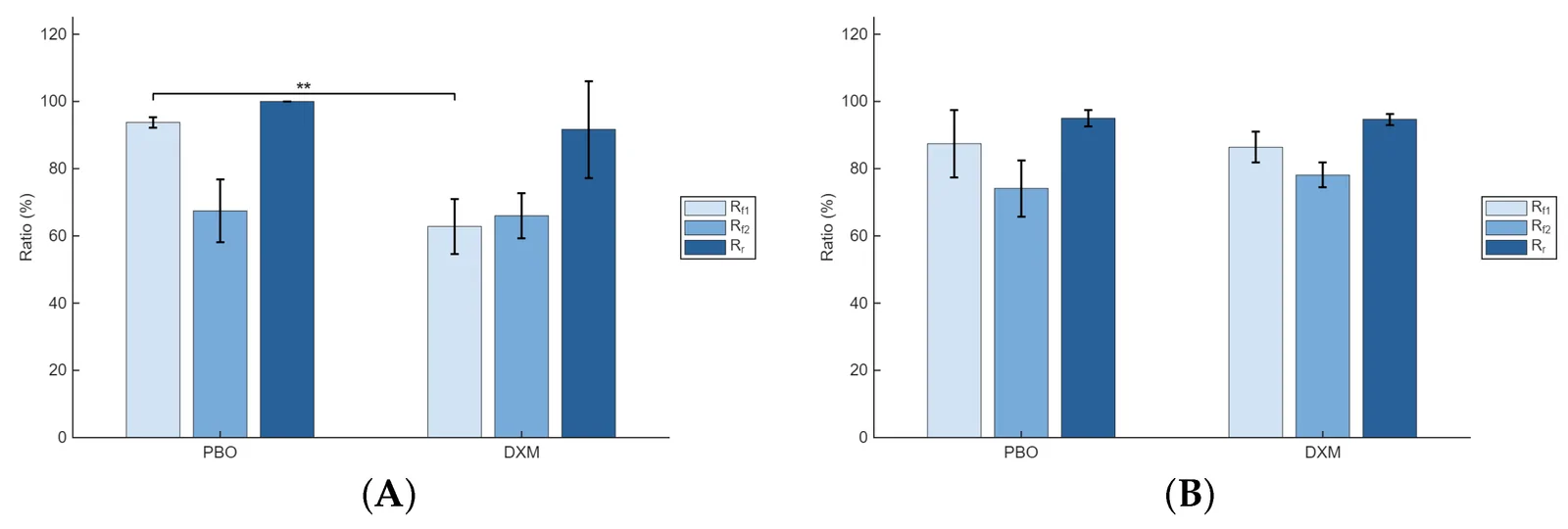
Shape fixity ratios after freezing ($R_{f1}$%) and drying ($R_{f2}$%), and shape recovery ratio ($R_{r}$%) of placebo (PBO) and DXM-loaded (DXM) ES (**A**) and DIW (**B**) scaffolds. Data are reported as mean ± standard deviation (n=3 replicates). Statistical comparisons between PBO and DXM scaffolds were performed using an unpaired two-tailed Student’s *t*-test. ** p<0.01 for ES $R_{f1}$; all other comparisons were not significant.

The results are reported in Figure 3. For ES scaffolds, PBO samples exhibited $R_{f1}$% values close to 95%, whereas DXM-loaded scaffolds showed significantly lower values, around 63% (p=0.0029). After drying, $R_{f2}$% values decreased and reached comparable levels for both PBP and DXM scaffolds, ranging between approximately 66 and 67%, respectively. Despite the different initial shape fixation behavior, both ES scaffold formulations exhibited high shape recovery efficiencies, with $R_{r}$% values above 90%.

Similarly, DIW scaffolds showed high shape fixity after freezing, with $R_{f1}$ values between approximately 85 and 90%. After drying, $R_{f2}$ values remained between 75 and 80% for both formulations, indicating a lower reduction in shape fixity during the drying step and suggesting improved stability of the programmed temporary configuration. High shape recovery was also observed for DIW scaffolds, with $R_{r}$% values above 90% in both PBO- and DXM-loaded samples.

Overall, all scaffold formulations demonstrated effective shape-memory behavior during the investigated single programming–recovery cycle, characterized by good temporary shape fixation (>60%) and high recovery (>90%) of the original geometry after thermal stimulation.

### 3.3. Dexamethasone Quantification

The amount of DXM loaded into ES and DIW scaffolds was quantified by HPLC. The extraction procedure showed an average recovery efficiency of 97.3±3.2%, confirming the reliability of the liquid–liquid extraction method for DXM quantification, as shown in Table 3.

ES scaffolds exhibited high DXM loading efficiencies above 93% under both SMT and NO SMT conditions, with values of 93.36 ± 8.91% for ES DXM SMT and 94.05 ± 1.57% for ES DXM NO SMT, respectively. DXM loading efficiencies for DIW scaffolds were 93.50 ± 6.29% and 82.73 ± 5.22% for DIW DXM SMT and DIW DXM NO SMT scaffolds, respectively. Overall, all formulations showed high drug loading capability and no significant loading efficiency differences after SMT, confirming the suitability of both fabrication approaches for DXM incorporation. Moreover, the preservation of high loading efficiencies after shape-memory programming indicates that the thermal treatment did not induce significant drug loss during scaffold processing.

### 3.4. In Vitro Drug Release

**Figure 4:**
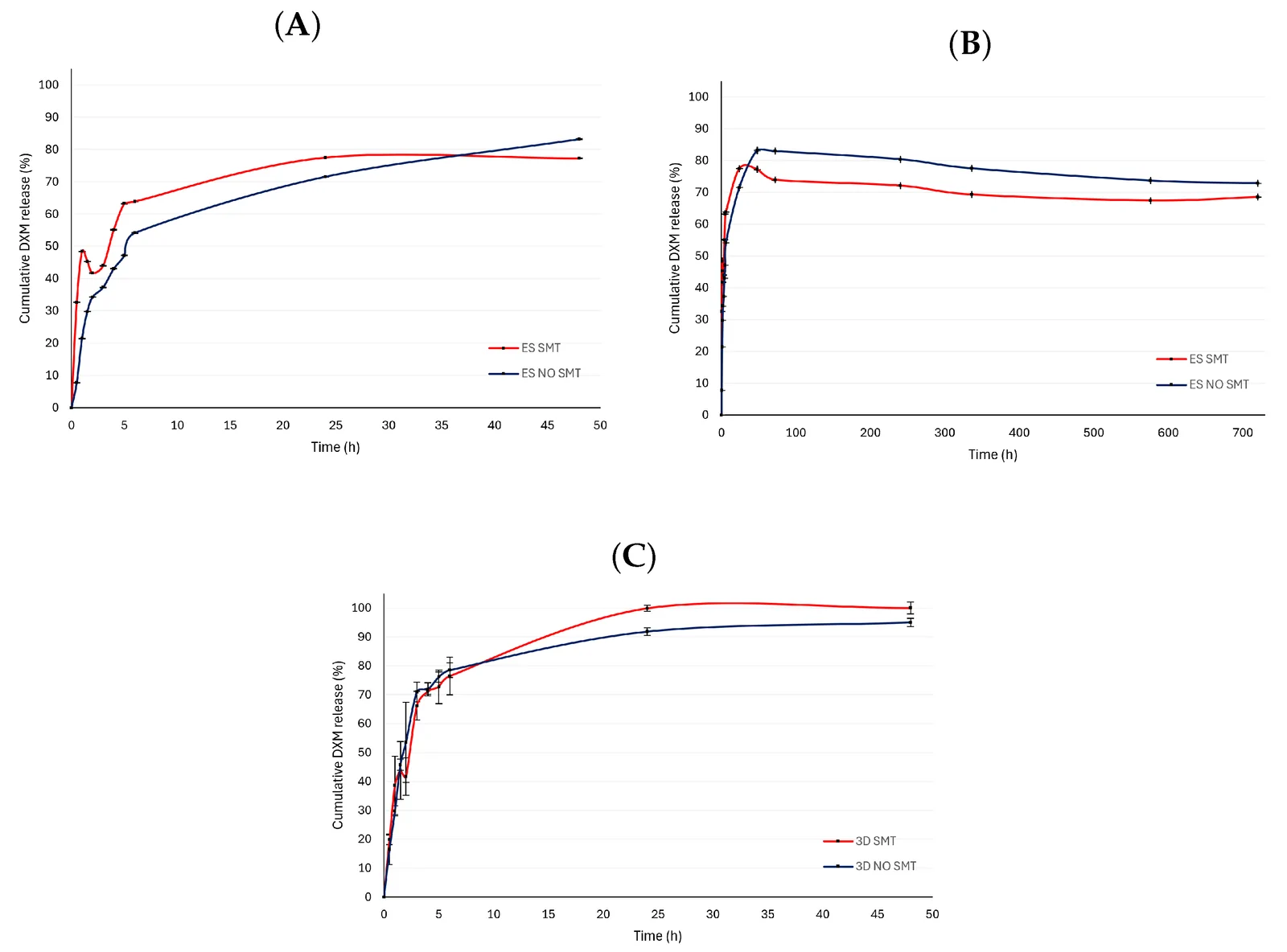
Cumulative DXM release profiles from ES and DIW scaffolds in 0.1 M HEPES buffer at 37 °C. (**A**) DXM release from ES scaffolds in the first 48 h; (**B**) long-term DXM release from ES scaffolds over 30 days; (**C**) DXM release from DIW scaffolds during the first 48 h. Data are expressed as mean ± SD.

The in vitro release profiles of DXM-loaded ES and DIW scaffolds were evaluated in 0.1 M HEPES buffer at 37 °C. Cumulative DXM release profiles in the initial 48 h are shown for the scaffolds obtained by both fabrication techniques, while the long-term 30-day release profile is reported only for ES scaffolds, since DIW samples achieved complete DXM release within the first 48 h (Figure 4).

ES scaffolds showed distinct release kinetics depending on SMT. ES DXM SMT scaffolds exhibited a faster initial release compared with ES DXM NO SMT, reaching approximately 40 % of cumulative DXM release within the first hour and 80 % within 24 h. Conversely, ES DXM NO SMT scaffolds showed a slower release rate during the first hours, reaching 80 % cumulative release within 48 h. After 48 h, both ES formulations reached a stable plateau, and no further DXM release was observed over the 30-day incubation.

For DIW scaffolds, both SMT and NO SMT samples showed a pronounced burst release in the first 24 h. DIW DXM SMT scaffolds reached approximately complete DXM release within 24 h, whereas DIW DXM NO SMT scaffolds released more than 90 % of the loaded drug within 24 h and achieved complete release within 48 h.

Overall, DIW scaffolds showed faster and more complete DXM release compared with ES scaffolds, while ES samples displayed slow DXM release behavior, achieving a maximum of approximately 80% drug release. This incomplete release can be attributed to the dense nanofibrous architecture of ES matrices, where a fraction of DXM may remain entrapped within the polymeric fibers due to the rapid solvent evaporation occurring during the electrospinning process. Moreover, the hydrophobic nature of the PLA/PCL copolymer may limit aqueous medium penetration, reducing drug diffusion from the inner fiber regions and resulting in partial retention of the incorporated drug.

To gain further insight into the mechanisms governing DXM release, the experimental data were fitted to different mathematical models, including zero-order, first-order, Higuchi, and Korsmeyer–Peppas equations. The kinetic parameters and corresponding coefficients of determination ($R^{2}$) are summarized in Table 4.

The kinetic analysis showed that the Higuchi model provided the best fit for ES SMT ($R^{2}=0.816$) and DIW SMT ($R^{2}=0.933$) scaffolds, indicating that DXM release was predominantly governed by diffusion through the polymeric matrix. Conversely, the Korsmeyer–Peppas model best described the release profiles of ES NO SMT ($R^{2}=0.895$) and DIW NO SMT ($R^{2}=0.994$). The corresponding release exponents (n=0.685 and 0.872, respectively) suggest an anomalous transport mechanism, in which drug diffusion is accompanied by polymer relaxation or structural rearrangement.

### 3.5. Wettability

**Figure 5:**
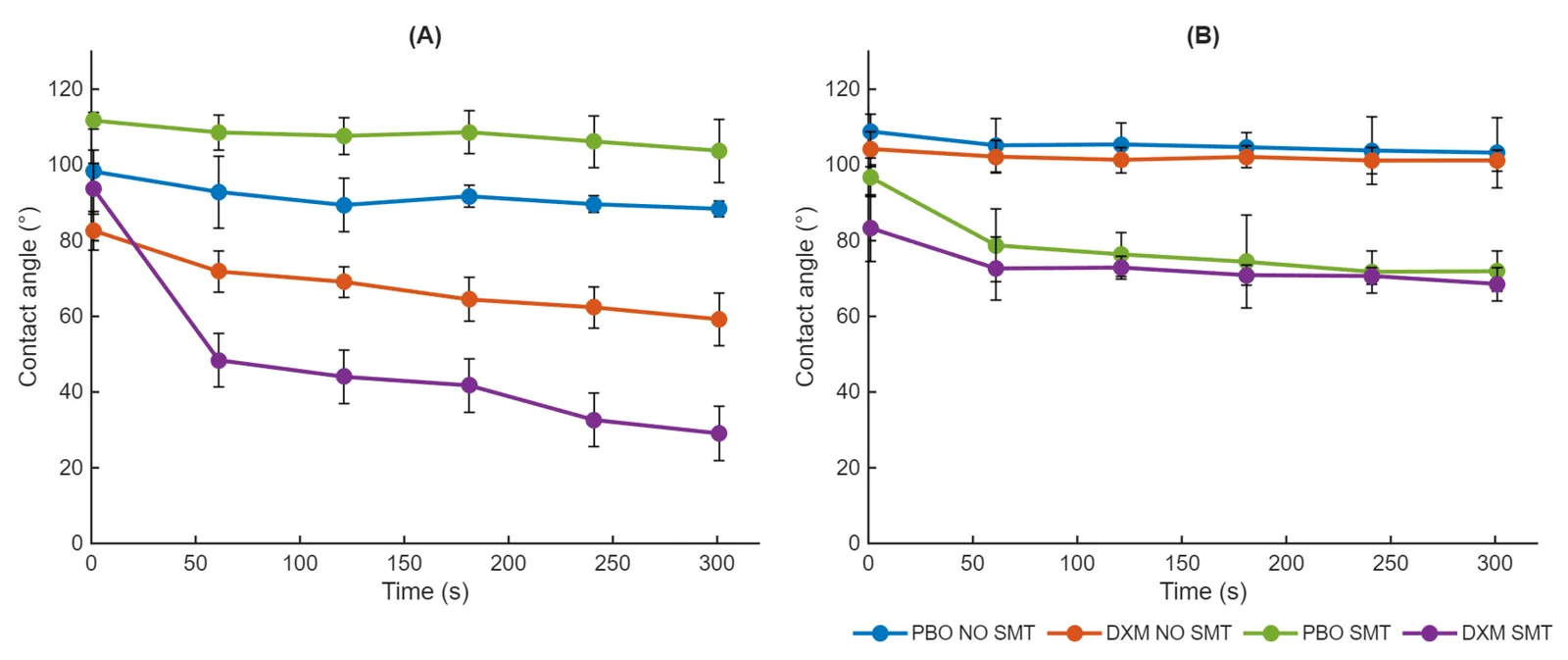
Time-dependent evolution of apparent contact angle values measured in HEPES buffer for (**A**) electrospun and (**B**) DIW PLA/PCL scaffolds. Measurements were performed on placebo (PBO) and dexamethasone-loaded (DXM) scaffolds before (NO SMT) and after shape-memory treatment (SMT). Contact angle measurements were recorded for 300 s following droplet deposition and are presented as mean ± SD (n=3).

Scaffold wettability was evaluated by time-dependent sessile-drop contact angle measurements in HEPES, PBS, and DMEM. Since comparable trends were observed in all investigated media, only the results obtained in HEPES, selected as the reference medium for the drug-release studies, are reported in the manuscript (Figure 5). The corresponding measurements performed in PBS and DMEM are provided in the Supplementary Material S1.4 (Figures S5 and S6).

ES PBO scaffolds exhibited contact angle values above $98^{\circ }$ at the starting time point and maintained relatively high values throughout the observation time (Figure 5A). In contrast, DXM-loaded ES scaffolds displayed lower initial contact angle values and a more pronounced decrease over time, indicating enhanced wettability. The greatest reduction was observed for ES DXM SMT, whose contact angle decreased from $93.74\pm 16.81^{\circ }$ at 0 min to $29.06\pm 7.09^{\circ }$ after 5 min, highlighting the combined influence of drug loading and shape-memory treatment on scaffold surface properties.

Both PBO- and DXM-loaded DIW scaffold formulations generally exhibited initial contact angle values above $83^{\circ }$ (Figure 5B). Untreated scaffolds maintained relatively stable contact angle values throughout the experiment, whereas shape-memory-treated samples showed a greater decrease over time. In particular, DIW PBO SMT decreased from $96.73\pm 5.00^{\circ }$ to $71.91\pm 15.40^{\circ }$, while DIW DXM SMT decreased from $83.33\pm 18.78^{\circ }$ to $68.55\pm 14.38^{\circ }$ after 6 min.

Overall, contact angle measurements demonstrated that scaffold wettability was strongly influenced by scaffold manufacturing technique, formulation composition, and shape-memory treatment. Among all investigated formulations, ES DXM SMT exhibited the largest reduction in contact angle values, suggesting a greater affinity for aqueous environments and potentially improved fluid penetration within the fibrous scaffold architecture. Similar wettability trends were observed in PBS and DMEM (Figures S1 and S2).

### 3.6. Mechanical Properties

The mechanical properties of ES and DIW scaffolds were evaluated by uniaxial tensile testing. The main mechanical parameters extracted from the engineering stress–strain curves were elongation at break ($\varepsilon_{b}$), maximum tensile stress ($\sigma_{\max}$), and Young’s modulus (*E*). The obtained values are summarized in Table 5.

ES scaffolds generally exhibited higher elongation at break and tensile strength than their DIW counterparts. Shape-memory treatment affected the mechanical behavior of ES scaffolds more markedly than that of DIW scaffolds. In particular, DXM-loaded ES scaffolds showed a reduction in both tensile strength and stiffness following thermal programming, with $\sigma_{\max}$ decreasing from 16.46±3.13 MPa to 7.12±1.05 MPa and Young’s modulus decreasing from 18.05±1.04 MPa to 5.04±0.87 MPa.

In contrast, DIW scaffolds exhibited lower tensile strength overall, with all formulations showing maximum tensile stress values below 2 MPa. Elongation at break ranged from 144.13±64.55% to 242.60±98.21%, while Young’s modulus values ranged from 4.29±1.05 MPa to 8.04±2.16 MPa. Compared with ES scaffolds, the effect of shape-memory treatment on the mechanical properties of DIW formulations appeared less pronounced.

Overall, the results indicate that scaffold architecture significantly influenced mechanical performance, with ES matrices exhibiting greater tensile resistance and deformability than the corresponding DIW structures.

### 3.7. Thermal Properties

The thermal properties of pristine PLA/PCL powder and scaffold formulations were evaluated by differential scanning calorimetry. $T_{g}$, cold crystallization peak temperature ($T_{cc}$), cold crystallization enthalpy ($\Delta H_{cc}$), melting peak temperature ($T_{m}$), and melting enthalpy ($\Delta H_{m}$) are reported in Table 6.

Pristine PLA/PCL powder showed a $T_{g}$ of 33.02 °C, a crystallization peak at 108.58 °C, and a melting peak at 157.68 °C. Scaffold processing did not have a significant effect on $T_{g}$, resulting in slight variations in $T_{g}$, with values ranging from 33.12 °C to 38.58 °C among the different formulations.

ES scaffolds showed crystallization peak temperatures ranging from 108.32 °C to 120.17 °C, while DIW scaffolds displayed lower crystallization peak temperatures, ranging from 84.15 °C to 105.95 °C. Melting peak temperatures remained within a narrow range for all samples, from 158.38 °C to 160.58 °C.

Crystallization and melting enthalpy of the pristine polymer are similar, indicating that the copolymer is mostly amorphous and confirming what has already been reported in the literature [16]. Among the tested scaffolds, DIW DXM SMT showed the highest crystallization and melting enthalpy values, with $\Delta H_{cc}=29.10$ J·g^−1^ and $\Delta H_{m}=31.77$ J·g^−1^, respectively. These values are very close to each other and to the $\Delta H_{cc}$ and $\Delta H_{m}$ of the pristine polymer, indicating that the copolymer solid state did not change during the DIW fabrication process and that the polymer remained almost amorphous.

ES DXM scaffolds showed lower enthalpy values, particularly ES DXM NO SMT, which displayed $\Delta H_{cc}=5.23$ J·g^−1^ and $\Delta H_{m}=12.29$ J·g^−1^, where the higher melting enthalpy relative to the cold-crystallization enthalpy suggests the presence of a greater pre-existing ordered fraction in the electrospun DXM-loaded scaffold. Interestingly, this phenomenon is evident only for ES DXM samples both before and after SMT.

Summarizing these results, it can be stated that: the copolymer is mostly amorphous; the DIW fabrication process does not change the polymer solid state, which remains almost amorphous even when supplemented with DXM; electrospinning of the polymer/DXM solution appears to modify the solid-state organization of the copolymer, while electrospinning has no effect on the solid state of the plain polymer. The higher crystallinity of the ES DXM samples might be a factor contributing to the slower release of DXM from the ES matrices compared with the DIW ones [36].

Literature on this topic is quite controversial. While the effect of electrospinning on the solid state of semicrystalline polymers is recognized [37], literature on DIW performed through a solvent-based deposition process at room temperature is still scarce, basically reporting that the crystallinity of PLA/PCL blends decreases because rapid solvent evaporation quenches the polymer chains [38].

### 3.8. Morphological Characterization by SEM

**Figure 6:**
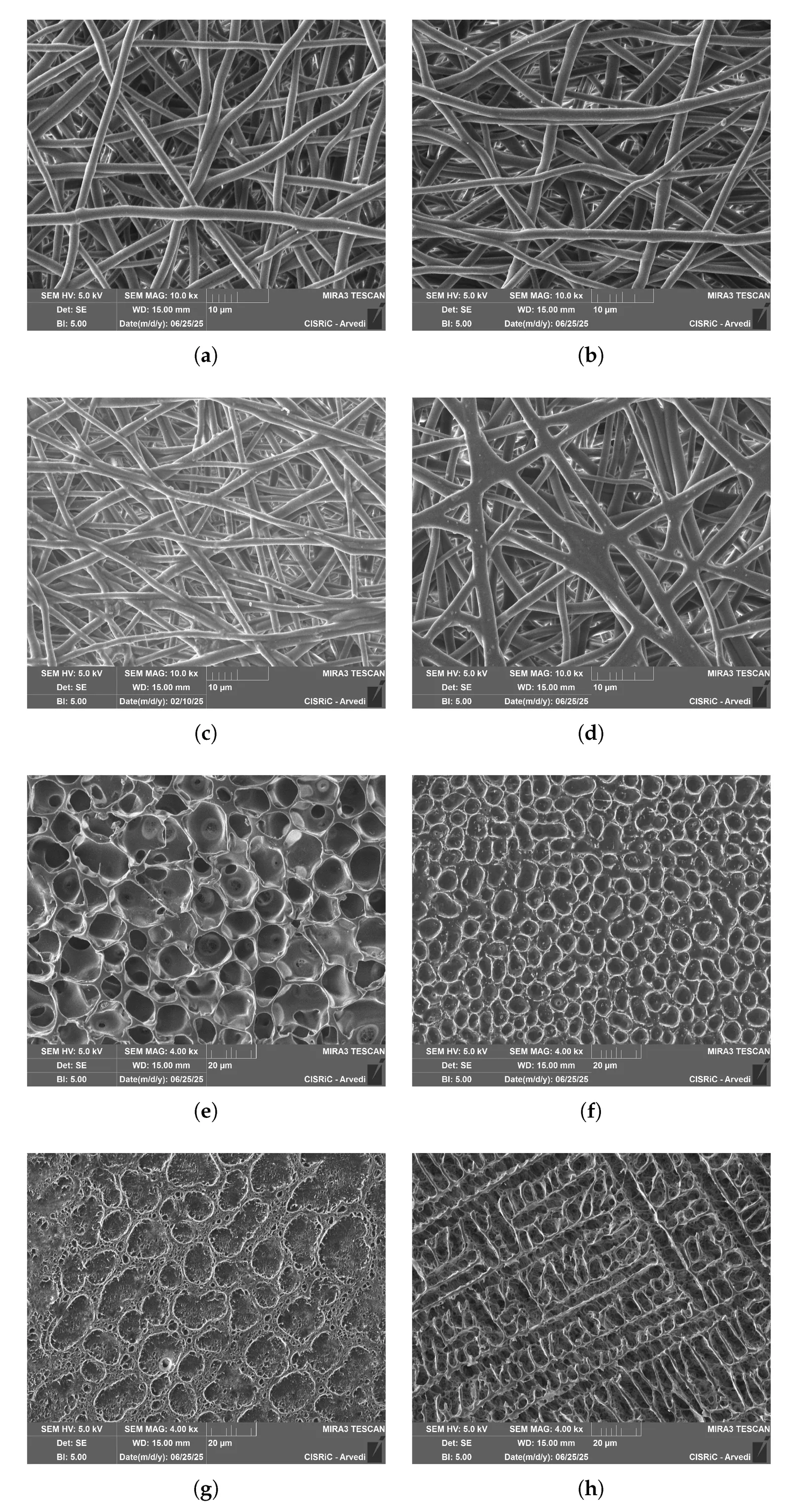
Representative SEM micrographs of ES and DIW PLA/PCL scaffolds before and after SMT. (**a**) ES DXM-loaded scaffold before SMT, (**b**) ES DXM-loaded scaffold after SMT, (**c**) ES PBO scaffold before SMT, (**d**) ES PBO scaffold after SMT, (**e**) DIW DXM-loaded scaffold before SMT, (**f**) DIW DXM-loaded scaffold after SMT, (**g**) DIW PBO scaffold before SMT, and (**h**) DIW PBO scaffold after SMT. Scale bars are reported in each micrograph.

SEM analysis was performed to evaluate the surface morphology and microporous structure of ES and DIW scaffolds before and after SMT. Representative SEM micrographs of ES and DIW scaffolds are shown in Figure 6, respectively, while the quantitative morphological parameters obtained from image analysis are reported in Table 7. In this analysis, pore diameter and porosity refer to surface micropores observed in the SEM micrographs, mainly associated with solvent removal and freeze-drying, rather than to the macroscopic designed pores of the DIW architecture.

ES scaffolds displayed a randomly oriented fibrous morphology in all analyzed conditions. The fibrous network was preserved after SMT, with no evident disruption of the scaffold microstructure. Quantitative analysis showed comparable mean fiber diameter among ES formulations, ranging from 0.309±0.06μm to 0.317±0.07μm. DXM-loaded ES scaffolds showed porosity values of 22.37±0.08% and 22.68±3.32% before and after SMT, respectively, with corresponding pore diameters of 1.83±0.01μm and 1.88±0.06μm. PBO ES scaffolds showed a porosity of 10.26±2.53% before SMT, which increased to 16.80±1.50% after SMT. Similarly, the mean pore diameter increased from 1.42±0.07μm to 1.81±0.04μm after SMT.

DIW scaffolds showed a continuous polymeric structure with surface microporosity visible in SEM micrographs. Compared with ES scaffolds, DIW samples exhibited higher surface porosity values. In DIW DXM scaffolds, porosity was 45.40±9.10% before SMT and 44.37±7.14% after SMT, with mean pore diameters of 2.36±0.61μm and 2.17±0.74μm, respectively. In DIW PBO scaffolds, porosity increased from 26.35±13.80% before SMT to 29.99±10.00% after SMT, whereas the mean pore diameter decreased from 2.37±1.51μm to 1.54±0.40μm.

Overall, SEM image analysis showed that SMT did not markedly alter ES fiber diameter, while differences among scaffold groups were mainly observed in surface microporosity and pore diameter.

## 4. Discussion

The present study provides a systematic comparison of ES and DIW for the fabrication of shape-memory PLA/PCL scaffolds. The novelty of this work does not rely on the individual use of either fabrication technique, both of which are already established, but on the direct evaluation of how two markedly different processing routes affect the multifunctional performance of the same PLA/PCL-based platform. PBO and DXM scaffolds were compared in terms of morphology, thermal properties, wettability, mechanical behavior, shape-memory performance, drug-loading efficiency, and release kinetics. ES and DIW processing parameters were deliberately optimized to obtain constructs with comparable weight (11.34 ± 1.20–11.97 ± 0.55 mg) and closely related sub-millimetric thickness, ensuring a reliable baseline for evaluating the influence of manufacturing-induced structural differences. Therefore, the main scientific contribution of the study lies in demonstrating that the fabrication route can be used as a design parameter to modulate the properties of drug-loaded shape-memory scaffolds prepared from the same polymeric platform. A further distinctive aspect of the present study is the application of the same specifically developed shape-memory programming approach to both ES and DIW architectures. This approach, based on a patented method for the production of shape-memory constructs [39], enabled the direct comparison of manufacturing-dependent differences in temporary-shape fixation, stability, and thermally triggered recovery under physiologically relevant conditions.

Morphological characterization confirmed that ES and DIW printing generated two significantly different scaffold architectures. ES produced nonwoven matrices composed of randomly oriented submicrometric fibers, closely resembling ECM-like fibrous structures. Importantly, fiber morphology was preserved independently of DXM incorporation and SMT application, with average fiber diameters remaining stable between 0.309 ± 0.06 and 0.317 ± 0.07 μm. This suggests that the ES parameters were the dominant factors controlling fiber formation, while drug loading and subsequent thermal programming did not compromise the nanofibrous organization [40,41]. Although fiber diameter remained unchanged, differences were detected in the surface void network. DXM-loaded ES scaffolds showed increased apparent surface porosity compared with PBO counterparts, suggesting that drug incorporation may influence fiber packing and scaffold microstructure. However, the preservation of the overall fibrous morphology indicates that DXM incorporation was compatible with the ES process.

Conversely, DIW printing produced continuous polymeric structures characterized by a more open architecture and higher surface porosity. This is consistent with previous studies demonstrating that extrusion-based printing enables the fabrication of scaffolds with controlled macroporous architectures, where filament arrangement and printing parameters directly determine the final pore network [42,43]. The final printed constructs maintained thickness values between 0.10 ± 0.01 and 0.15 ± 0.03 mm, comparable to ES scaffolds. The microporous features observed on the printed structures are likely associated with solvent removal during the freeze-drying process, which contributed to the formation of surface voids.

Thermal characterization showed that all scaffold formulations maintained $T_{g}$ values between approximately 33 and 39 °C. These values were close to the $T_{g}$ of pristine PLA/PCL powder and indicate that neither fabrication nor DXM incorporation markedly shifted the thermal transition involved in activation of the shape-memory response. The preservation of a transition interval close to physiological temperature is consistent with the thermo-responsive behavior previously reported for this PLA/PCL copolymer [16]. Overall, SEM analysis confirmed that SMT did not induce major structural alterations in either scaffold type, demonstrating that both architectures were able to withstand thermal programming [44].

All scaffold formulations maintained $T_{g}$ values between 33 and 39 °C, indicating that neither the fabrication process nor DXM loading markedly shifted the thermal transition involved in the shape-memory response of PLA/PCL. More relevant differences emerged from the comparison between melting and cold-crystallization enthalpies. For all DIW formulations, the difference $\Delta H_{m}-\Delta H_{cc}$ remained relatively limited (1.40–2.67 (J·g^−1^)), and similarly low values were observed for ES PBO scaffolds (approximately 0–2.40 (J·g^−1^). In contrast, ES DXM scaffolds showed a more pronounced difference, reaching 7.06 (J·g^−1^) before SMT and 3.79 (J·g^−1^) after SMT. Since a larger excess of melting enthalpy over cold-crystallization enthalpy is consistent with the presence of crystalline domains already formed before the DSC heating scan, these results suggest a greater pre-existing ordered fraction specifically in ES DXM scaffolds. Importantly, this trend was not observed in ES PBO samples, indicating that it cannot be attributed to ES alone.

The observed behavior may therefore result from the combined effects of ES and DXM incorporation. During ES, the strong elongational forces acting on the polymer jet promote chain stretching and orientation, while solvent evaporation progressively reduces chain mobility and fixes the molecular organization reached during fiber formation. The final solid-state arrangement depends on the balance between these two phenomena: chain orientation can favor the development of ordered domains, whereas rapid solidification can limit the time available for further molecular rearrangement. In the present study, the fact that the larger enthalpy difference appeared mainly in DXM-loaded ES scaffolds suggests that the presence of DXM influenced this balance during fiber formation, possibly by modifying polymer–drug interactions, chain mobility, or crystallization kinetics. Changes in the thermal and cold-crystallization behavior of electrospun polyester fibers following DXM incorporation have also been reported previously [45].

Nevertheless, the present DSC data do not allow the specific role of DXM to be identified. In particular, it cannot be established whether the drug directly promoted nucleation, modified polymer-chain packing, or affected the solidification process through its interactions with the polymer and solvent system. Therefore, the observed enthalpy differences should be regarded as evidence of a different solid-state organization of ES DXM scaffolds rather than as definitive proof of a specific crystallization mechanism. Complementary structural techniques, such as X-ray diffraction, would be required to confirm and quantitatively characterize these differences.

This different polymer organization may also contribute to the release behavior observed for ES DXM scaffolds. A greater crystalline or ordered fraction generally reduces polymer-chain mobility and creates a less accessible diffusional environment for incorporated molecules, potentially slowing drug transport through the polymer matrix. This mechanism is consistent with the slower and incomplete DXM release observed from ES scaffolds, which reached a plateau of approximately 80%. However, crystallinity alone cannot account for the release profile. Fiber packing, drug localization within the fibers, scaffold wettability, polymer–drug interactions, and accessibility of the release medium are also likely to contribute to the differences observed between ES and DIW scaffolds.

Accordingly, all formulations displayed excellent recovery ability, with shape recovery ratios exceeding 90% after exposure to physiological temperature. The activation temperature close to physiological conditions represents a key requirement for minimally invasive SMP-based devices, allowing temporary shape fixation before implantation and recovery after exposure to body temperature [14]. This suggests that the continuous printed structure provides greater geometrical stability during temporary shape retention, whereas the flexible fibrous ES network undergoes greater rearrangement during programming and drying.

Shape-memory performance was, however, evaluated only after a single programming–recovery cycle. The present findings therefore demonstrate the feasibility of temporary-shape fixation and thermally triggered recovery, but do not establish the repeatability or cyclic durability of the scaffolds. Repeated programming may induce progressive chain relaxation, fiber or strut rearrangement, permanent deformation, or changes in shape-fixity and shape-recovery ratios. Future investigations should therefore assess scaffold performance over multiple consecutive cycles and determine whether the observed recovery ability is maintained after repeated activation.

The different scaffold architectures also influenced their interaction with aqueous environments. Contact angle measurements showed a progressive decrease over time, particularly for ES DXM SMT scaffolds, indicating enhanced wettability after drug incorporation and thermal programming. The nanofibrous organization of ES scaffolds likely promoted liquid spreading due to the high available surface area, whereas DIW scaffolds exhibited a more moderate time-dependent variation. These results indicate that wettability is strongly affected not only by material composition but also by the architecture generated during processing. Because the investigated scaffolds exhibited porous and topographically heterogeneous surfaces, the observed time-dependent contact-angle behavior should be interpreted as the combined result of surface chemistry, apparent surface porosity, capillary penetration, liquid absorption, and local topography, rather than as a direct measure of surface roughness alone. In this context, complementary atomic force microscopy (AFM) analyses would be useful in future studies to independently characterize local surface roughness and better clarify its contribution to scaffold wettability. Direct gravimetric water-uptake and dimensional-swelling measurements were not performed in the present study; therefore, the time-dependent contact-angle data should not be interpreted as quantitative measurements of equilibrium water absorption or swelling. Dedicated hydration studies will be required to characterize these properties in future investigations.

These architecture-dependent differences directly translated into distinct DXM release profiles. Despite their different manufacturing routes, both approaches achieved high DXM incorporation, with loading efficiencies above 93% for ES scaffolds and between 82.73 ± 5.22% and 93.50 ± 6.29% for DIW scaffolds NO SMT and SMT, respectively. Moreover, SMT did not significantly affect loading efficiency, confirming that the thermal programming procedure preserved the incorporated drug.

Release kinetics differed markedly between the two scaffold architectures. DIW scaffolds provided faster and almost complete DXM release within 24–48 h. Their more accessible porous structure likely facilitated penetration of the release medium and reduced the effective diffusion distance [46]. Conversely, ES scaffolds showed a more gradual release profile and reached a plateau at approximately 80% of the loaded drug. This partial release may reflect the combined effects of the dense interconnected fibrous network, reduced accessibility of DXM located within or between the fibers, drug–polymer interactions, and the greater ordered fraction suggested by DSC. Similar architecture-dependent release behavior has been reported for electrospun drug-loaded matrices, in which fiber organization and drug distribution affect the accessibility and diffusion of the incorporated molecules [47,48].

These mechanistic interpretations should nevertheless be considered with caution because the spatial distribution and physical state of DXM within the scaffolds were not directly investigated. Moreover, release experiments were performed in HEPES buffer under controlled and simplified in vitro conditions. This approach enabled comparison of the two scaffold architectures while minimizing variability associated with the release medium, but it does not fully reproduce the biochemical complexity of physiological environments. Proteins, enzymes, lipids, ionic components, and local pH variations may modify surface wetting, DXM solubility, drug–polymer interactions, and mass transport through the scaffold. In addition, progressive degradation of PLA/PCL may alter the pore network and diffusion pathways over time. Since scaffold degradation was not evaluated in the present study, the reported profiles should be interpreted as comparative in vitro release data rather than as direct predictors of in vivo drug-release behavior.

Mechanical characterization further confirmed the strong relationship between scaffold architecture and functional response. ES scaffolds displayed superior mechanical properties compared with DIW constructs due to their interconnected fibrous network. In particular, ES DXM NO SMT exhibited the highest mechanical performance, with an elongation at break of 288.10 ± 40.59%, tensile strength of 16.46 ± 3.13 MPa, and Young’s modulus of 18.05 ± 1.04 MPa. This behavior can be attributed to the ability of randomly oriented fibers to progressively rearrange and redistribute stress during tensile deformation [49]. After SMT, ES DXM scaffolds maintained high elongation (320.27 ± 79.21%) but showed reduced tensile strength (7.12 ± 1.05 MPa) and stiffness (5.04 ± 0.87 MPa), indicating that thermal programming affected the mechanical response of the fibrous network. In contrast, DIW scaffolds exhibited lower tensile strength values (<2 MPa) but showed smaller variations after SMT, suggesting that the printed architecture maintained a more stable mechanical response following programming.

Uniaxial tensile testing was selected as a standardized preliminary method for comparing the two scaffold architectures. However, this test alone does not reproduce the complex mechanical conditions that may occur after implantation. Cyclic loading would be required to assess fatigue resistance and mechanical hysteresis, while stress-relaxation, creep, compression, and dynamic mechanical analysis could provide further information on viscoelastic and temperature-dependent behavior. The present mechanical results should therefore be interpreted as an initial comparative characterization rather than as a complete validation of scaffold performance for a specific anatomical application.

Overall, these results demonstrate that fabrication technique is a key parameter controlling the multifunctional behavior of PLA/PCL-based 4D scaffolds. ES generates mechanically robust and highly deformable nanofibrous matrices capable of providing sustained DXM release, whereas DIW printing enables geometrically stable structures characterized by efficient shape fixation and faster drug availability. Therefore, rather than identifying one superior fabrication method, this study highlights how manufacturing technique can be exploited as a design tool to tailor biodegradable 4D scaffolds according to specific requirements for minimally invasive tissue engineering and localized drug delivery.

Several limitations must nevertheless be acknowledged. No biological experiments were conducted on the specific scaffolds investigated in the present study, and the current work should therefore be regarded as a physicochemical and functional proof-of-concept rather than as biological validation of scaffold suitability for tissue engineering. Nevertheless, preliminary biological evidence is already available for the same PLA/PCL 70:30 material platform. In a previous study, electrospun PLA/PCL 70:30 shape-memory scaffolds were shown to support mesenchymal stem cell viability and proliferation following shape-memory programming [50]. These findings provide preliminary biological support for the use of this material platform in regenerative applications. Scaffold architecture, processing history, possible residual solvents, DXM incorporation, and shape-memory treatment may all influence cell–material interactions. Cytocompatibility, cell adhesion, proliferation, inflammatory response, and preservation of the biological activity of released DXM will therefore require dedicated investigation before the biomedical relevance of these systems can be established.

A further limitation is the use of three replicates per condition. This sample size was considered appropriate for a preliminary material-characterization study and enabled the evaluation of mean values and experimental variability. However, it provides limited statistical power, particularly for identifying moderate differences between formulations. Except for the predefined comparisons of shape-memory parameters, the differences observed in the other characterizations should therefore be interpreted primarily as descriptive trends. Confirmation using larger sample sizes, independent fabrication batches, repeated shape-memory cycles, additional mechanical tests, and more physiologically representative release and biological models will be required to establish the reproducibility and translational potential of the developed scaffolds.

## 5. Conclusions

This study demonstrated that fabrication technique plays a decisive role in determining the structural and functional performance of shape-memory PLA/PCL scaffolds, even when the same copolymer composition is employed. Although ES and DIW produced scaffolds with comparable overall mass and thickness, the two techniques generated markedly different architectures, which, in turn, influenced wettability, mechanical behavior, shape-memory performance, and drug release kinetics.

The present study also highlights the importance of selecting the fabrication technique according to the intended biomedical application. ES scaffolds may be advantageous when a biomimetic fibrous architecture and enhanced mechanical compliance are required, whereas DIW scaffolds may be preferable when architectural control and efficient drug release are the primary objectives.

Nevertheless, some limitations of the study should be acknowledged and retained as future perspectives. Mechanical characterization was restricted to uniaxial tensile testing, while drug release was evaluated under simplified in vitro conditions. Moreover, although previous studies have already demonstrated the biocompatibility of the PLA/PCL copolymer, the biological responses associated with the electrospun and DIW-printed scaffolds were not investigated in this work. Future studies should include rheological and printability analyses, evaluation of scaffold performance under physiologically relevant conditions, and in vitro biological assessment to establish the relationship between scaffold architecture, cellular response, and therapeutic efficacy.

Overall, the results demonstrate that, even when generating structurally distinct yet functionally competent platforms, these fabrication techniques offer complementary opportunities for future applications in minimally invasive regenerative medicine and localized drug delivery.

## Figures and Tables

**Table 1:** DIW parameters used for scaffold fabrication.

Parameter	Value
Length	30 mm
Width	15 mm
Height	0.30 mm
Pore size	0.30 mm
Layer height	0.15 mm
Flow speed	0.8 mm/s
Infill speed	7 mm/s
Perimetral speed	10 mm/s
Perimeters	1

**Table 2:** Weight and thickness of ES and DIW PBO and DXM-loaded scaffolds.

Scaffold Composition	Weight (mg)	Thickness (mm)
ES PBO	11.94±0.60	0.06±0.01
ES DXM	11.34±1.20	0.06±0.01
DIW PBO	11.54±0.14	0.10±0.01
DIW DXM	11.97±0.55	0.15±0.03

**Table 3:** Theoretical and experimental DXM content and loading efficiency of ES and DIW DXM-loaded scaffolds with and without SMT.

Scaffold Composition	Theoretical DXM (mg)	Experimental DXM (mg)	Loading Efficiency (%)
ES DXM SMT	0.075	0.068±0.006	93.36±8.91
ES DXM NO SMT	0.075	0.069±0.001	94.05±1.57
DIW DXM SMT	0.074	0.069±0.009	93.50±6.29
DIW DXM NO SMT	0.074	0.061±0.007	82.73±5.22

**Table 4:** Kinetic fitting parameters and corresponding best-fit model for the DXM release profiles.

Formulation	Zero-Order		First-Order		Higuchi		Korsmeyer–Peppas	
	$k_{0}$	$R^{2}$	$k_{1}$(h^−1^)	$R^{2}$	$k_{H}$	$R^{2}$	n	$R^{2}$
ES SMT	0.0416	0.681	0.0416	0.815	10.05	0.816	0.173	0.563
ES NO SMT	0.0431	0.678	0.0431	0.841	13.87	0.860	0.685	0.895
DIW SMT	0.2233	0.871	0.2233	0.932	32.37	0.933	0.566	0.841
DIW NO SMT	0.2491	0.833	0.2491	0.919	36.20	0.925	0.872	0.994

**Table 5:** Mechanical properties of ES and DIW scaffolds under uniaxial tensile testing.

Scaffold Composition	$\varepsilon_{b}$ (%)	$\sigma_{\max}$ (MPa)	*E* (MPa)
ES DXM SMT	320.27±79.21	7.12±1.05	5.04±0.87
ES DXM NO SMT	288.10±40.59	16.46±3.13	18.05±1.04
ES PBO SMT	150.90±85.84	4.06±2.97	8.29±6.88
ES PBO NO SMT	306.07±47.51	2.89±2.41	6.95±2.39
DIW DXM SMT	144.13±64.55	1.93±0.77	8.04±2.16
DIW DXM NO SMT	214.93±70.29	1.89±0.22	6.53±1.63
DIW PBO SMT	196.27±33.67	1.88±0.39	5.26±1.59
DIW PBO NO SMT	242.60±98.21	1.41±0.49	4.29±1.05

**Table 6:** DSC parameters of pristine PLA/PCL powder and ES and DIW scaffold formulations before and after SMT.

Sample	$T_{g}$ (°C)	$T_{\mathrm{cc}}$ (°C)	$\Delta H_{\mathrm{cc}}$ ($J\cdot g^{-1}$)	$T_{m}$ (°C)	$\Delta H_{m}$ ($J\cdot g^{-1}$)
DIW PBO SMT	36.24	97.35	18.27	159.31	19.90
DIW PBO NO SMT	38.58	105.95	10.32	159.74	12.48
DIW DXM SMT	34.71	84.94	29.10	158.86	31.77
DIW DXM NO SMT	33.94	84.15	15.17	158.66	16.57
ES PBO SMT	37.43	108.32	12.51	160.58	14.91
ES PBO NO SMT	34.62	113.07	17.79	159.54	16.97
ES DXM SMT	35.89	120.17	9.14	159.15	12.93
ES DXM NO SMT	33.12	119.49	5.23	158.38	12.29
PLA/PCL powder	33.02	108.58	22.89	157.68	22.11

**Table 7:** SEM-derived morphological parameters of ES and DIW scaffolds before and after SMT. Pore diameter and porosity refer to surface micropores observed in SEM micrographs.

Scaffold Composition	Fiber Diameter (μm)	Surface Porosity (%)	Surface Pore Diameter (μm)
ES DXM NO SMT	0.313±0.07	22.37±0.08	1.83±0.01
ES DXM SMT	0.309±0.06	22.68±3.32	1.88±0.06
ES PBO NO SMT	0.317±0.07	10.26±2.53	1.42±0.07
ES PBO SMT	0.312±0.07	16.80±1.50	1.81±0.04
DIW DXM NO SMT	–	45.40±9.10	2.36±0.61
DIW DXM SMT	–	44.37±7.14	2.17±0.74
DIW PBO NO SMT	–	26.35±13.80	2.37±1.51
DIW PBO SMT	–	29.99±10.00	1.54±0.40

## Data Availability

The data presented in this study are available upon reasonable request from the corresponding author.

## Supplementary Materials

The following supporting information can be downloaded at: https://www.mdpi.com/article/10.3390/pharmaceutics18091084/s1, Table S1: Summary of the main 3D-printing parameter combinations evaluated during scaffold optimization; Table S2: Influence of PLA/PCL solution concentration on the dimensional properties of DIW scaffolds; Figure S1: Kinetic modelling of DXM release from ES SMT scaffolds over the selected fitting interval; Figure S2: Kinetic modelling of DXM release from ES NO SMT scaffolds over the selected fitting interval; Figure S3: Kinetic modelling of DXM release from DIW SMT scaffolds over the selected fitting interval; Figure S4: Kinetic modelling of DXM release from DIW NO SMT scaffolds over the selected fitting interval; Figure S5: Time-dependent evolution of static contact angle values measured in PBS for ES and DIW PLA/PCL scaffolds; Figure S6: Time-dependent evolution of static contact angle values measured in DMEM for ES and DIW PLA/PCL scaffolds. Supplementary MATLAB scripts for shape-memory analysis, DXM release kinetic modelling, mechanical data analysis, SEM-based porosity and pore-size analysis, and electrospun fiber-diameter analysis are also provided.

## Abbreviations

The following abbreviations are used in this manuscript:

3D	Three-dimensional
4D	Four-dimensional
BHT	Butylated hydroxytoluene
CAD	Computer-aided design
CCD	Charge-coupled device
DCM	Dichloromethane
DIW	Direct Ink Writing
DMEM	Dulbecco’s Modified Eagle Medium
DMF	N,N-dimethylformamide
DSC	Differential Scanning Calorimetry
DXM	Dexamethasone
ECM	Extracellular Matrix
ES	Electrospun
HEPES	4-(2-hydroxyethyl)-1-piperazineethanesulfonic acid
HPLC	High-Performance Liquid Chromatography
IVF	Injection Volume Filling
LE	Loading Efficiency
PBS	Phosphate-Buffered Saline
PBO	Placebo
PCL	Polycaprolactone
PLA	Polylactide
PLA/PCL	Poly(L-lactide-co-caprolactone)
Rf	Shape Fixity Ratio
Rf_1_	Shape Fixity Ratio after Freezing
Rf_2_	Shape Fixity Ratio after Freeze-Drying
Rr	Shape Recovery Ratio
SEM	Scanning Electron Microscopy
SMT	Shape Memory Treatment
Tc	Crystallization Temperature
Tg	Glass Transition Temperature
THF	Tetrahydrofuran
Tm	Melting Temperature
ULT	Ultra-Low Temperature
UV	Ultraviolet
