# Supplementary material for: Toward 4D Biomaterials: Comparing Electrospun and 3D-Printed Shape-Memory Scaffolds
Source: Pharmaceutics. 2026 Aug 28;18(9):1084. doi: 10.3390/pharmaceutics18091084 (PMC13610966; doi:10.3390/pharmaceutics18091084)
Supplement: Supplementary file 1 [file pharmaceutics-18-01084-s001.zip › pharmaceutics-4471150-supplementary.pdf]

Supplementary Materials

Supplementary Section S1.1 Optimization of 3D-Printing Parameters

The primary objective of this study was to directly compare ES and DIW PLA/PCL scaffolds by minimizing macroscopic differences between the two systems. Since ES scaffolds had already been optimized in terms of dimensions, weight, and thickness, they were used as the reference platform for the development of DIW constructs. Therefore, ink formulation and printing parameters were systematically optimized to fabricate DIW scaffolds with the same macroscopic dimensions (3 × 1.5 cm) and comparable weight and thickness to the ES counterparts, allowing a reliable evaluation of the influence of fabrication-induced architecture on scaffold performance.

The optimization process was initially performed using a PLA/PCL polymeric solution at a concentration of 15% (w/v). Printing parameters were optimized using the Regemat3D software, which allows independent modification of the main process variables, including the number of deposited layers, pore size, extrusion flow speed, layer height, infill speed, and printing speed. Several combinations of these parameters were investigated in a stepwise manner, progressively refining the printing conditions to obtain a reproducible scaffold geometry with dimensional features comparable to the ES reference scaffolds.

The main printing conditions explored during the optimization process are summarized in Table S1. Among the tested configurations, Test 4 provided the best compromise between printability and macroscopic scaffold characteristics, producing constructs with an average thickness of approximately 0.11 mm and dimensions comparable to the electrospun counterparts. Consequently, this printing configuration was selected as the optimized set of parameters for the subsequent development of DIW scaffolds.

Supplementary Table S1: Summary of the main 3D-printing parameter combinations evaluated during scaffold optimization. The average scaffold weight obtained under each printing condition is also reported. Test 4 was selected as the optimal printing configuration.

**Printing parameter**	**Test 1**	**Test 2**	**Test 3**	**Test 4**
Number of layers	4	2	1	2
Pore size (mm)	0.30	0.30	0.30	0.30
Flow speed (mm/s)	0.20	0.20	0.20	0.08
Layer height (mm)	0.30	0.30	0.30	0.15
Infill speed (mm/s)	14	13	10	7
Print speed (mm/s)	15	14	12	10
Scaffold weight (mg)	43.0 ± 2.0	25.0 ± 3.0	18.0 ± 1.0	16.0 ± 0.2

Although the optimization of printing parameters using the 15% (w/v) PLA/PCL solution allowed the fabrication of scaffolds with suitable geometry and thickness, the resulting constructs still exhibited higher weights compared with the ES reference scaffolds, even when using the lowest printable settings.

Therefore, after defining the optimal printing configuration, different PLA/PCL solution concentrations (15, 10, and 8 wt%) were investigated to further reduce scaffold weight while maintaining the optimized geometry and dimensional reproducibility. As expected, decreasing the polymer concentration progressively reduced the scaffold weight.

Scaffolds printed using the 15 wt% formulation exhibited an average weight of approximately 15.0 ± 0.2 mg, whereas the 10 wt% formulation yielded scaffolds weighing approximately 13.0 ± 0.3 mg. The lowest concentration (8 wt%) produced scaffolds with an average weight of approximately 11.0 ± 0.1 mg, closely matching the weight of the electrospun scaffolds prepared in this study.

Based on the results reported in supplementary table S2, the 8 wt% PLA/PCL solution was selected as the final ink formulation for the fabrication of all DIW scaffolds used throughout the experimental work, as it provided constructs with comparable dimensions and weight to the ES reference scaffolds while preserving the previously optimized printing geometry.

Supplementary Table S2: Influence of PLA/PCL solution concentration on the dimensional properties of DIW scaffolds. Scaffold weight and thickness values are reported as mean ± SD ($n = 3$).

**PLA/PCL**	**Weight (mg)**	**Thickness (mm)**
10 wt%	13.0 ± 0.3	0.13 ± 0.01
8 wt%	11.0 ± 0.1	0.11 ± 0.03

Overall, this optimization strategy ensured that the final electrospun and DIW scaffolds exhibited comparable dimensions and mass, thereby allowing the observed differences in morphology, mechanical behavior, wettability, shape-memory performance, and drug release to be primarily attributed to the fabrication technique rather than to differences in scaffold geometry.

Supplementary Section S1.2 MATLAB Script for Shape Memory Analysis

The shape-memory parameters, namely the shape fixity ratio after freezing (R_{f1}), the shape fixity ratio after vacuum drying (R_{f2}), and the shape recovery ratio (R_r), were calculated using a custom MATLAB script. The algorithm performs image calibration using a reference ruler, extracts scaffold geometry from manually selected regions of interest, and calculates the shape-memory indices according to the equations reported in the main manuscript.

```
function [Rf1, Rf2, Rr] = calculate_Rf_Rr( ...
    img_initial_path, img_Rf1_path, img_Rf2_path, ...
    img_recovery_path, ruler_distance_mm)
% calculate_Rf_Rr
% Calculates the shape fixity ratio after freezing (Rf1),
% the shape fixity ratio after vacuum drying (Rf2),
% and the shape recovery ratio (Rr) from scaffold images.
% Inputs:
%   img_initial_path  - image of the initial flat scaffold
%   img_Rf1_path      - image of the scaffold after freezing
```

```
%   img_Rf2_path      - image of the scaffold after vacuum drying
%   img_recovery_path - image of the scaffold after recovery
%   ruler_distance_mm - real distance between two ruler marks [mm]
% Outputs:
%   Rf1 - shape fixity ratio after freezing [%]
%   Rf2 - shape fixity ratio after vacuum drying [%]
%   Rr  - shape recovery ratio [%]


%% Initial scaffold


img_initial = imread(img_initial_path);
imshow(img_initial);
title('Initial scaffold: select two ruler marks');


[x_r, y_r] = ginput(2);
px_ruler = sqrt((x_r(2)-x_r(1))^2 + ...
                (y_r(2)-y_r(1))^2);
px_per_mm = px_ruler / ruler_distance_mm;


imshow(img_initial);
title('Initial scaffold: select scaffold contour');
mask_initial = roipoly;


props_initial = regionprops(mask_initial, 'Area', 'Perimeter');


area_initial_mm2 = props_initial.Area / (px_per_mm^2);
perimeter_initial_mm = props_initial.Perimeter / px_per_mm;


%% Shape fixity after freezing (Rf1)


img_Rf1 = imread(img_Rf1_path);
```

```
imshow(img_Rf1);
title('Rf1: select two ruler marks');

[x_r, y_r] = ginput(2);
px_ruler = sqrt((x_r(2)-x_r(1))^2 + ...
                (y_r(2)-y_r(1))^2);
px_per_mm = px_ruler / ruler_distance_mm;

imshow(img_Rf1);
title('Rf1: select rolled scaffold diameter');

[x_d, y_d] = ginput(2);
px_diameter = sqrt((x_d(2)-x_d(1))^2 + ...
                   (y_d(2)-y_d(1))^2);
diameter_Rf1_mm = px_diameter / px_per_mm;

D_ref = 1.5; % mandrel diameter [mm]

Rf1 = (D_ref / diameter_Rf1_mm) * 100;

%% Shape fixity after vacuum drying (Rf2)

img_Rf2 = imread(img_Rf2_path);
imshow(img_Rf2);
title('Rf2: select two ruler marks');

[x_r, y_r] = ginput(2);
px_ruler = sqrt((x_r(2)-x_r(1))^2 + ...
                (y_r(2)-y_r(1))^2);
px_per_mm = px_ruler / ruler_distance_mm;

imshow(img_Rf2);
```

```
title('Rf2: select rolled scaffold diameter');

[x_d, y_d] = ginput(2);
px_diameter = sqrt((x_d(2)-x_d(1))^2 + ...
                   (y_d(2)-y_d(1))^2);
diameter_Rf2_mm = px_diameter / px_per_mm;

Rf2 = (D_ref / diameter_Rf2_mm) * 100;

%% Shape recovery ratio (Rr)

img_recovery = imread(img_recovery_path);
imshow(img_recovery);
title('Recovered scaffold: select two ruler marks');

[x_r, y_r] = ginput(2);
px_ruler = sqrt((x_r(2)-x_r(1))^2 + ...
                (y_r(2)-y_r(1))^2);
px_per_mm = px_ruler / ruler_distance_mm;

imshow(img_recovery);
title('Recovered scaffold: select scaffold contour');
mask_recovery = roipoly;

props_recovery = regionprops(mask_recovery, 'Area', 'Perimeter');

area_recovery_mm2 = props_recovery.Area / (px_per_mm^2);
perimeter_recovery_mm = props_recovery.Perimeter / px_per_mm;

Rr_area = (area_recovery_mm2 / area_initial_mm2) * 100;
Rr_perimeter = (perimeter_recovery_mm / perimeter_initial_mm) * 100;
```

```
Rr = mean([Rr_area, Rr_perimeter]);

%% Quality control

if Rf1 < 0 || Rf1 > 100
    warning('Rf1 outside the expected range of 0-100%.');
end

if Rf2 < 0 || Rf2 > 100
    warning('Rf2 outside the expected range of 0-100%.');
end

if Rr < 0 || Rr > 100
    warning('Rr outside the expected range of 0-100%.');
end

end

% Example:
%
% [Rf1, Rf2, Rr] = calculate_Rf_Rr( ...
%     'initial.jpg', ...
%     'frozen.jpg', ...
%     'vacuum_dried.jpg', ...
%     'recovered.jpg', ...
%     10);
```

Listing S1: MATLAB script used for the calculation of shape-memory parameters from scaffold images.

Supplementary Section S1.3 Drug Release Kinetic Modelling

The MATLAB script used for DXM release kinetic modelling is reported below. The corresponding graphical outputs obtained for each formulation are shown in Figures S1–S4.

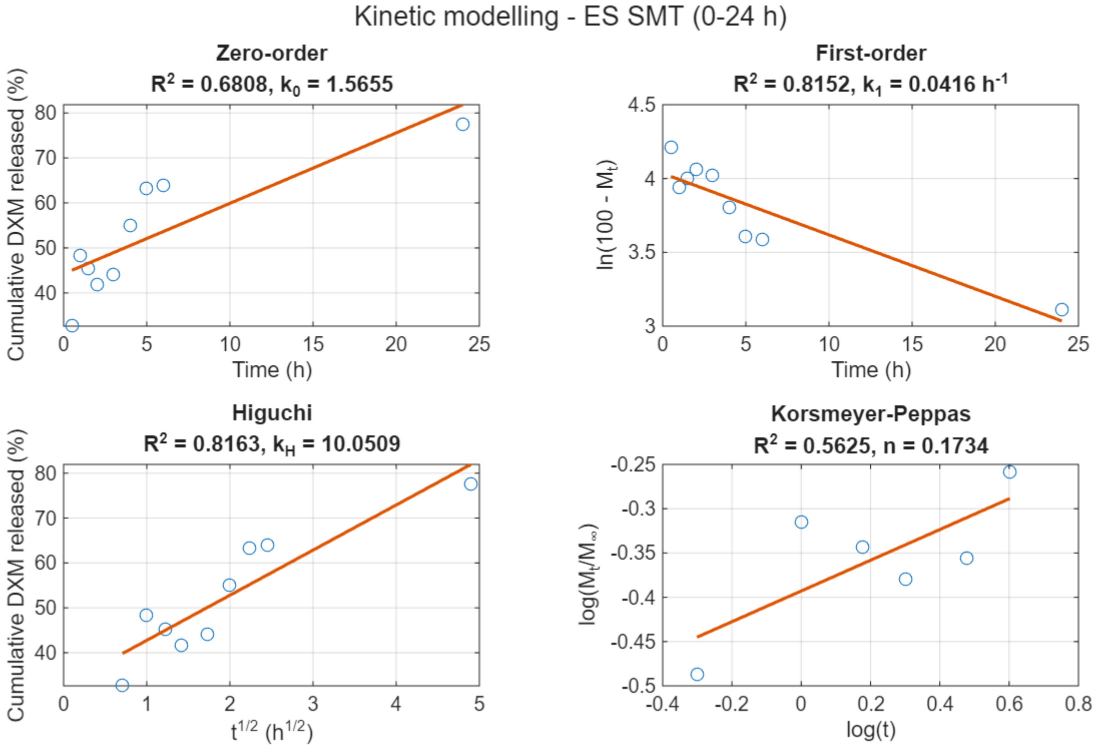

Supplementary Figure S1: Kinetic modelling of DXM release from ES SMT scaffolds over the selected fitting interval. Zero-order, first-order, Higuchi, and Korsmeyer–Peppas models are reported.

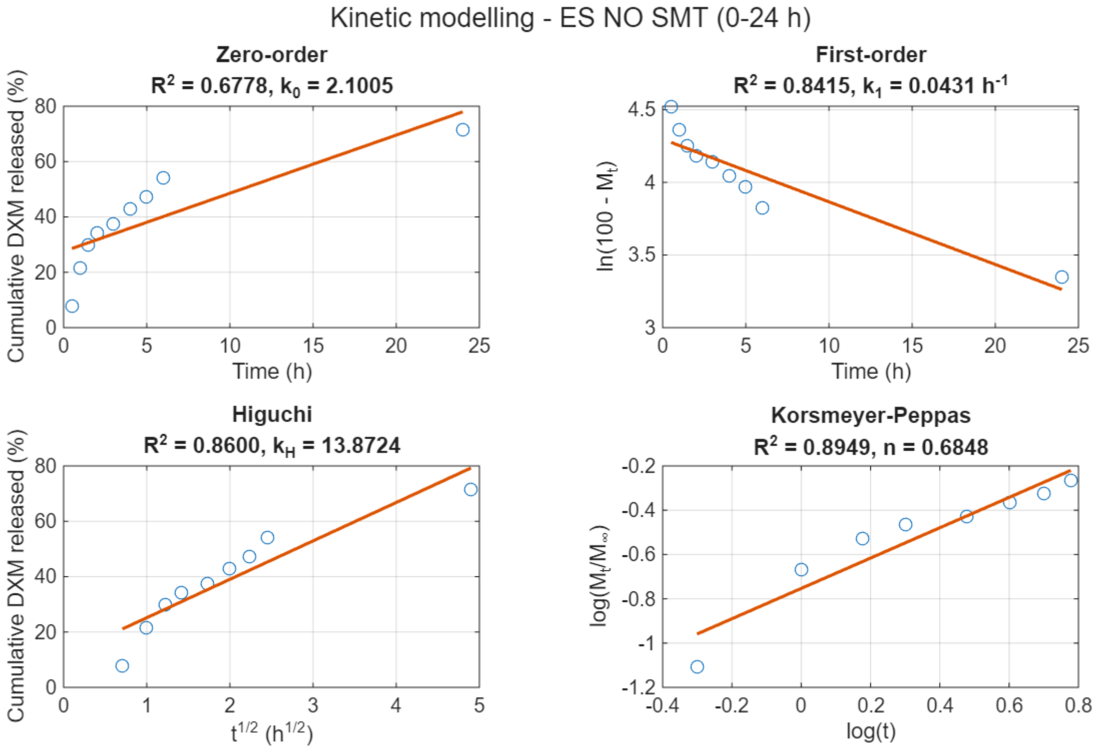

Supplementary Figure S2: Kinetic modelling of DXM release from ES NO SMT scaffolds over the selected fitting interval. Zero-order, first-order, Higuchi, and Korsmeyer–Peppas models are reported.

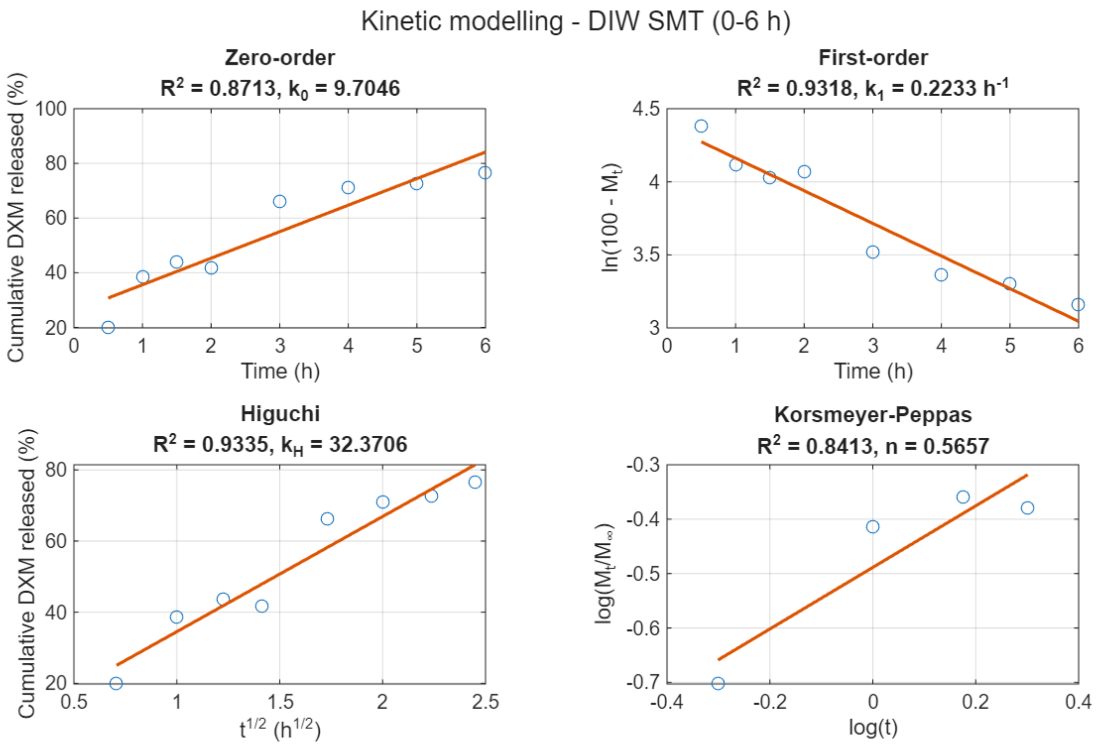

Supplementary Figure S3: Kinetic modelling of DXM release from DIW SMT scaffolds over the selected fitting interval. Zero-order, first-order, Higuchi, and Korsmeyer–Peppas models are reported.

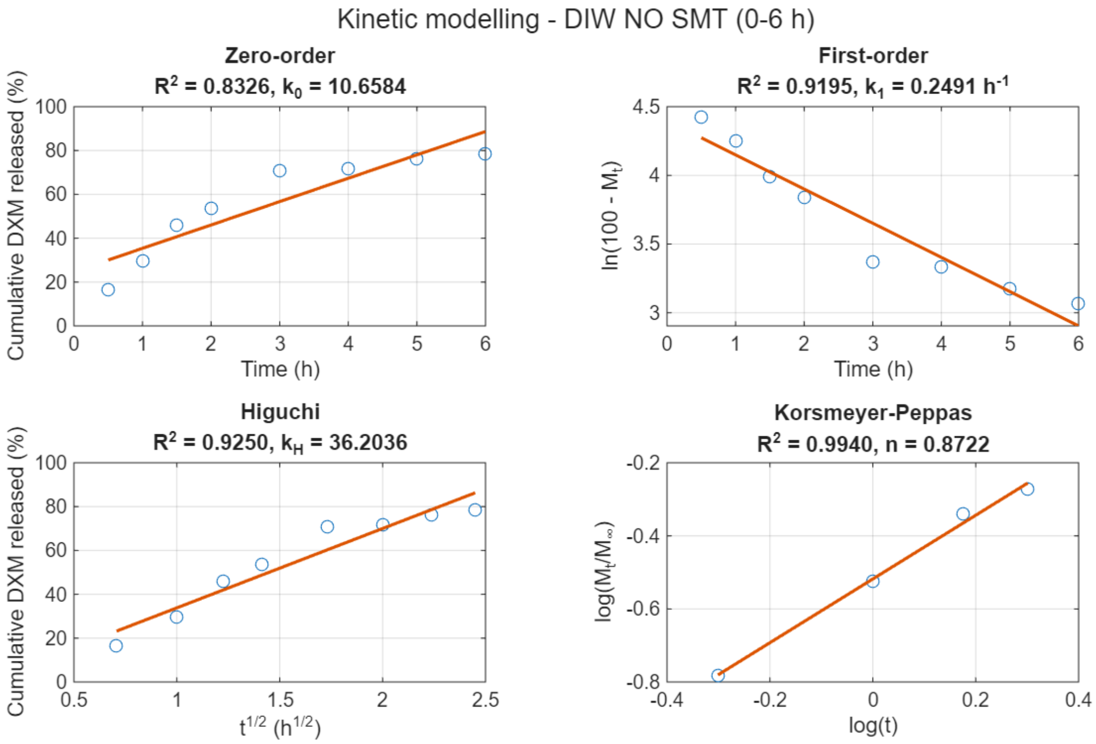

Supplementary Figure S4: Kinetic modelling of DXM release from DIW NO SMT scaffolds over the selected fitting interval. Zero-order, first-order, Higuchi, and Korsmeyer–Peppas models are reported.

```
%% DXM release kinetic modelling
% Models: zero-order, first-order, Higuchi, Korsmeyer-Peppas
% Data expressed as cumulative DXM released (%)

clear; clc; close all;

%% Experimental data

t = [0 0.5 1 1.5 2 3 4 5 6 24 48 72 240 336 576 720]';

ES_SMT = [0 32.618 48.414 45.339 41.751 44.038 55.102 63.213 63.903 ...
          77.494 77.247 73.967 72.124 69.326 67.430 68.552]';

ES_NO_SMT = [0 7.7968 21.490 29.794 34.322 37.305 43.067 47.159 ...
             54.160 71.556 83.183 82.964 80.412 77.554 73.718 72.876]';

D3_SMT = [0 19.819 38.618 43.775 41.684 66.223 71.114 72.752 ...
          76.457 99.876 100 97.366 98.621 96.324 95.507 93.838]';

D3_NO_SMT = [0 16.514 29.827 45.762 53.496 70.914 71.863 76.109 ...
             78.467 91.816 94.978 94.468 98.185 96.746 97.287 97.000]';

datasets = {
    'ES SMT',     ES_SMT,     24;
    'ES NO SMT',  ES_NO_SMT,  24;
    'DIW SMT',     D3_SMT,      6;
    'DIW NO SMT',  D3_NO_SMT,   6
};

Results = table();

%% Kinetic modelling

for i = 1:size(datasets,1)

    name = datasets{i,1};
    Mt = datasets{i,2};
    tmax_fit = datasets{i,3};

    idx = t > 0 & t <= tmax_fit & Mt > 0 & Mt < 100;

    tf = t(idx);
    Mf = Mt(idx);

    %% Zero-order model

    [k0, q0, R2_zero, y_zero_fit] = fitLinear(tf, Mf);

    %% First-order model

    y_first = log(100 - Mf);

    [slope_first, q_first, R2_first, y_first_fit] = fitLinear(tf, y_first);

    k1 = -slope_first;

    %% Higuchi model

    x_higuchi = sqrt(tf);

    [kH, qH, R2_higuchi, y_higuchi_fit] = fitLinear(x_higuchi, Mf);

    %% Korsmeyer-Peppas model
```

```
idx_kp = Mf <= 60;

tf_kp = tf(idx_kp);
Mf_kp = Mf(idx_kp);

x_kp = log10(tf_kp);
y_kp = log10(Mf_kp/100);

[n, logkKP, R2_KP, y_kp_fit] = fitLinear(x_kp, y_kp);

kKP = 10^logkKP;

%% Best model identification

R2_all = [R2_zero, R2_first, R2_higuchi, R2_KP];

model_names = {'Zero-order', 'First-order', ...
               'Higuchi', 'Korsmeyer-Peppas'};

[~, best_idx] = max(R2_all);

best_model = model_names{best_idx};

%% Store results

Results = [Results; table( ...
    string(name), ...
    tmax_fit, ...
    k0, q0, R2_zero, ...
    k1, q_first, R2_first, ...
    kH, qH, R2_higuchi, ...
    kKP, n, R2_KP, ...
    string(best_model), ...
    'VariableNames', { ...
    'Formulation', 'Fitting_time_h', ...
    'k0', 'Zero_intercept', 'R2_zero', ...
    'k1_h_minus_1', 'First_intercept', 'R2_first', ...
    'kH', 'Higuchi_intercept', 'R2_Higuchi', ...
    'kKP', 'n', 'R2_KP', ...
    'Best_model'})];

%% Plot kinetic fits

figure('Name', name, 'Color', 'w');

tiledlayout(2,2);

nexttile;
plot(tf, Mf, 'o', 'MarkerSize', 6); hold on;
plot(tf, y_zero_fit, '-', 'LineWidth', 1.5);
xlabel('Time (h)');
ylabel('Cumulative DXM released (%)');
title(sprintf('Zero-order\nR^2 = %.4f, k_0 = %.4f', R2_zero, k0));
grid on;

nexttile;
plot(tf, y_first, 'o', 'MarkerSize', 6); hold on;
plot(tf, y_first_fit, '-', 'LineWidth', 1.5);
xlabel('Time (h)');
ylabel('ln(100 - M_t)');
title(sprintf('First-order\nR^2 = %.4f, k_1 = %.4f h^{-1}', ...
      R2_first, k1));
```

```
    grid on;

    nexttile;
    plot(x_higuchi, Mf, 'o', 'MarkerSize', 6); hold on;
    plot(x_higuchi, y_higuchi_fit, '-', 'LineWidth', 1.5);
    xlabel('t^{1/2} (h^{1/2})');
    ylabel('Cumulative DXM released (%)');
    title(sprintf('Higuchi\nR^2 = %.4f, k_H = %.4f', ...
          R2_higuchi, kH));
    grid on;

    nexttile;
    plot(x_kp, y_kp, 'o', 'MarkerSize', 6); hold on;
    plot(x_kp, y_kp_fit, '-', 'LineWidth', 1.5);
    xlabel('log(t)');
    ylabel('log(M_t/M_inf)');
    title(sprintf('Korsmeyer-Peppas\nR^2 = %.4f, n = %.4f', ...
          R2_KP, n));
    grid on;

    sgtitle(sprintf('Kinetic modelling - %s (0-%.0f h)', ...
            name, tmax_fit));

end

disp(Results);

%% Local function for linear regression

function [slope, intercept, R2, y_fit] = fitLinear(x, y)

    x = x(:);
    y = y(:);

    p = polyfit(x, y, 1);

    slope = p(1);
    intercept = p(2);

    y_fit = polyval(p, x);

    SS_res = sum((y - y_fit).^2);
    SS_tot = sum((y - mean(y)).^2);

    R2 = 1 - SS_res / SS_tot;

end
```

Listing S2: MATLAB script used for DXM release kinetic modelling.

Supplementary Section S1.4 Additional Contact Angle Measurements

To complement the wettability analysis presented in the main manuscript, additional static contact angle measurements performed in PBS and DMEM are reported below. As in the main text, measurements were carried out on ES and DIW PLA/PCL scaffolds, including PBO and DXM formulations before (NO SMT) and after shape-memory treatment (SMT). Contact angle values were monitored for 301 s following droplet deposition and are reported as mean ± SD ($n = 3$).

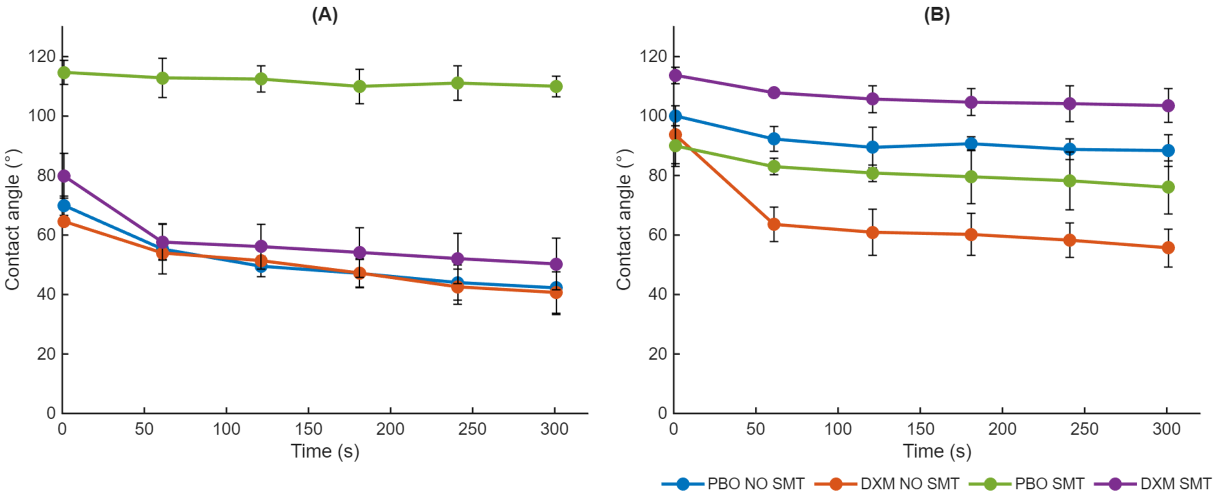

Supplementary Figure S5: Time-dependent evolution of static contact angle values measured in PBS for ES (A) and DIW (B) PLA/PCL scaffolds. Measurements were performed on placebo (PBO) and dexamethasone-loaded (DXM) scaffolds before (NO SMT) and after shape-memory treatment (SMT). Data are presented as mean ± SD ($n = 3$).

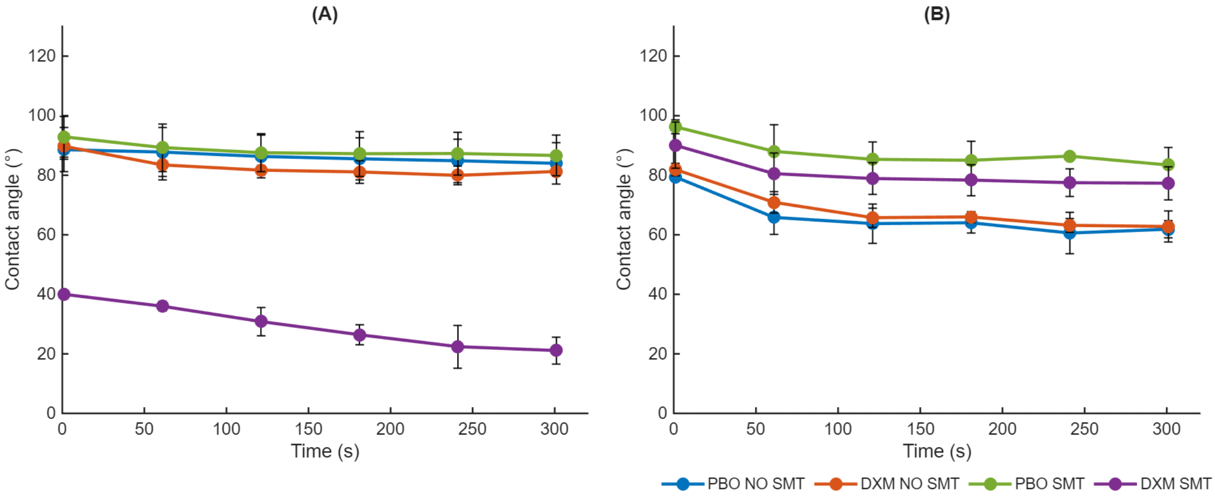

Supplementary Figure S6: Time-dependent evolution of static contact angle values measured in DMEM for ES (A) and DIW (B) PLA/PCL scaffolds. Measurements were performed on placebo (PBO) and dexamethasone-loaded (DXM) scaffolds before (NO SMT) and after shape-memory treatment (SMT). Data are presented as mean ± SD ($n = 3$).

Supplementary Section S1.5 MATLAB Script for Mechanical Data Analysis

The MATLAB script used for processing tensile testing data and extracting Young's modulus, maximum tensile stress, and elongation at break is reported below.

```
%% Mechanical data analysis
% Extraction of Young's modulus, maximum tensile stress, and elongation at break.
% The elastic region was identified using a linear fit with R2 >= 0.99
% and a minimum of 20 data points.

clear; clc; close all;

%% Common parameters

```

```
L0     = 10;    % initial gauge length [mm]
width  = 15;    % specimen width [mm]
R2_thr = 0.99;  % minimum R2 threshold for elastic fit
minPts = 20;    % minimum number of points for elastic fit

%% Sample information

% samples must be provided as a structure array with:
% samples(i).file = Excel file name
% samples(i).t    = specimen thickness [mm]
% samples(i).type = scaffold group
%
% Example:
% samples = [
%   struct('file','sample_1.xlsx','t',0.10,'type','DIW_PBO_NOSMT')
%   struct('file','sample_2.xlsx','t',0.12,'type','DIW_PBO_NOSMT')
% ];

%% Results table

N = numel(samples);

Results = table('Size',[N 5], ...
    'VariableTypes',{'string','string','double','double','double'}, ...
    'VariableNames',{'File','Group','E_MPa','SigmaMax_MPa','ElongPct'});

%% Loop over samples

for i = 1:N

    fn = samples(i).file;
    t  = samples(i).t;

    % Import force-displacement data
    T  = readtable(fn,'Sheet','Foglio2');
    F0 = T.Load_N_;
    D0 = T.Travel_mm_;

    % Automatic identification of the effective starting point
    dF = diff(F0);
    dD = diff(D0);

    Nwin = 5;

    idx0 = find(arrayfun(@(k) ...
        all(dF(k:k+Nwin-1)>0 & dD(k:k+Nwin-1)>0), ...
        1:(numel(dF)-Nwin+1)),1) + 1;

    if isempty(idx0)
        idx0 = 1;
    end

    F = F0(idx0:end) - F0(idx0);
    D = D0(idx0:end) - D0(idx0);

    % Trimming after maximum load
    [~,iMax] = max(F);

    iEnd = min(iMax+3,numel(F));

    F = F(1:iEnd);
    D = D(1:iEnd);

```

```
    % Engineering stress and strain
    A       = width * t;   % cross-sectional area [mm2]
    sigma   = F / A;       % engineering stress [MPa]
    epsilon = D / L0;      % engineering strain [-]

    % Identification of the linear elastic region
    bestK = minPts;

    for k = minPts:numel(epsilon)

        p = polyfit(epsilon(1:k),sigma(1:k),1);

        yhat = polyval(p,epsilon(1:k));

        R2 = 1 - sum((sigma(1:k)-yhat).^2) / ...
                 sum((sigma(1:k)-mean(sigma(1:k))).^2);

        if R2 >= R2_thr
            bestK = k;
        else
            break;
        end

    end

    % Mechanical parameters
    p_el = polyfit(epsilon(1:bestK),sigma(1:bestK),1);

    E        = p_el(1);
    sigmaMax = sigma(iMax);
    elongPct = epsilon(iMax) * 100;

    % Store results
    Results.File(i)         = string(fn);
    Results.Group(i)        = string(samples(i).type);
    Results.E_MPa(i)        = E;
    Results.SigmaMax_MPa(i) = sigmaMax;
    Results.ElongPct(i)     = elongPct;

end

%% Group statistics

groups = unique(Results.Group);
G = numel(groups);

meanE   = zeros(G,1);
stdE    = zeros(G,1);
meanSig = zeros(G,1);
stdSig  = zeros(G,1);
meanEl  = zeros(G,1);
stdEl   = zeros(G,1);

for j = 1:G

    idx = Results.Group == groups(j);

    meanE(j)   = mean(Results.E_MPa(idx));
    stdE(j)    = std(Results.E_MPa(idx));

    meanSig(j) = mean(Results.SigmaMax_MPa(idx));
    stdSig(j)  = std(Results.SigmaMax_MPa(idx));

```

```
    meanEl(j)  = mean(Results.ElongPct(idx));
    stdEl(j)   = std(Results.ElongPct(idx));

end

Statistics = table( ...
    groups, ...
    meanE, stdE, ...
    meanSig, stdSig, ...
    meanEl, stdEl, ...
    'VariableNames',{ ...
    'Group', ...
    'Mean_E_MPa','Std_E_MPa', ...
    'Mean_SigmaMax_MPa','Std_SigmaMax_MPa', ...
    'Mean_ElongPct','Std_ElongPct'});

disp(Results);
disp(Statistics);

%% Optional export to Excel

outFile = 'Mechanical_analysis_results.xlsx';

writetable(Results,outFile,'Sheet','RawData');
writetable(Statistics,outFile,'Sheet','Statistics');

%% Optional bar plots

plotMechanicalParameter(groups,meanE,stdE, ...
    'Young''s modulus','E [MPa]',[0 20]);

plotMechanicalParameter(groups,meanSig,stdSig, ...
    'Maximum tensile stress','\sigma_{max} [MPa]',[0 20]);

plotMechanicalParameter(groups,meanEl,stdEl, ...
    'Elongation at break','Elongation [%]',[0 500]);

%% Local function for bar plots

function plotMechanicalParameter(groups,meanValues,stdValues,plotTitle,yLabel,
    yLimits)

    figure('Color','w');
    hold on;

    b = bar(meanValues, ...
        'FaceColor',[0.20 0.45 0.70], ...
        'EdgeColor','none');

    x = b.XEndPoints;

    errorbar(x,meanValues,stdValues, ...
        'k', ...
        'LineStyle','none', ...
        'CapSize',10, ...
        'LineWidth',1.2);

    set(gca, ...
        'XTick',1:numel(groups), ...
        'XTickLabel',groups, ...
        'XTickLabelRotation',45, ...
        'TickLabelInterpreter','none', ...
        'FontSize',12, ...
```

```
        'LineWidth',1.1);

    ylabel(yLabel);
    title(plotTitle);

    ylim(yLimits);

    box off;
    grid off;

end
```

Listing S3: MATLAB script used for mechanical data processing.

Supplementary Section S1.6 MATLAB Script for SEM Porosity and Pore Size Analysis

The MATLAB script used for SEM image processing and quantitative analysis of surface porosity and pore diameter is reported below.

```
%% SEM porosity and pore size analysis
% Quantification of apparent surface porosity and equivalent pore diameter
% from SEM micrographs.

clear; clc; close all;

%% Input data

% filenames must contain the SEM images to be analyzed.
% threshold_factors can be adjusted for each image when required.

% Example:
%
% filenames = {
%     'sample_1.tif'
%     'sample_2.tif'
%     'sample_3.tif'
% };
%
% threshold_factors = [0.72 0.72 0.72];

%% Initialize results

nImages = length(filenames);

porosity_values = zeros(1,nImages);
mean_pore_diameters = zeros(1,nImages);

%% Scale calibration

sample_img = imread(filenames{1});

if size(sample_img,3) == 3
    sample_gray = rgb2gray(sample_img);
else
    sample_gray = sample_img;
end

figure;
imshow(sample_gray,[]);
title('Select the beginning and end of the scale bar');

```

```
[x,y] = ginput(2);
close;

real_length_um = input('Enter the real scale bar length [um]: ');

scale_bar_pixels = sqrt((x(2)-x(1))^2 + (y(2)-y(1))^2);
um_per_pixel = real_length_um / scale_bar_pixels;

fprintf('\nCalculated scale: %.4f um/pixel\n',um_per_pixel);

%% Image processing loop

for i = 1:nImages

    img = imread(filenames{i});

    if size(img,3) == 3
        img_gray = rgb2gray(img);
    else
        img_gray = img;
    end

    % Crop image to remove SEM scale bar and metadata
    [rows,~] = size(img_gray);
    img_crop = img_gray(1:round(0.85*rows),:);

    % Filtering and thresholding
    img_filtered = imgaussfilt(img_crop,1);

    threshold = graythresh(img_filtered) * threshold_factors(i);

    bw = imbinarize(img_filtered,threshold);

    % Invert binary image: pores = 1, polymer = 0
    bw = ~bw;

    %% Apparent porosity

    total_pixels = numel(bw);
    pore_pixels = sum(bw(:));

    porosity = (pore_pixels / total_pixels) * 100;

    porosity_values(i) = porosity;

    %% Equivalent pore diameter

    pores_clean = bwareaopen(bw,10);

    stats = regionprops(pores_clean,'Area');

    pore_areas_px = [stats.Area];

    if ~isempty(pore_areas_px)

        pore_diameters_um = ...
            2 * sqrt((pore_areas_px * (um_per_pixel^2)) / pi);

        mean_pore_diameters(i) = mean(pore_diameters_um);

    else

        mean_pore_diameters(i) = NaN;
```

```
    end

    %% Optional visualization

    figure;
    imshow(bw);
    title(sprintf( ...
        'Image %d: Porosity = %.2f%% | Mean pore diameter = %.2f um', ...
        i,porosity,mean_pore_diameters(i)));

end

%% Final statistics

mean_porosity = mean(porosity_values,'omitnan');
std_porosity  = std(porosity_values,'omitnan');

mean_diameter = mean(mean_pore_diameters,'omitnan');
std_diameter  = std(mean_pore_diameters,'omitnan');

fprintf('\nFinal results\n');
fprintf('Mean porosity: %.2f%%\n',mean_porosity);
fprintf('SD porosity: %.2f%%\n',std_porosity);
fprintf('Mean pore diameter: %.2f um\n',mean_diameter);
fprintf('SD pore diameter: %.2f um\n',std_diameter);
```

Listing S4: MATLAB script used for SEM-based porosity and pore diameter analysis.

Supplementary Section S1.7 Fiber Diameter Analysis of Electrospun Scaffolds

The MATLAB script used for SEM-based fiber diameter quantification of electrospun scaffolds is reported below.

```
%% SEM fiber diameter analysis
% Quantification of fiber diameter from SEM micrographs.
% Fibers are segmented by thresholding and analyzed by skeletonization
% and distance transform.

clear; clc; close all;

%% Input data

% img_folder defines the folder containing the SEM images.
% filenames contains the list of images to be analyzed.
%
% Example:
%
% img_folder = 'path_to_SEM_images';
%
% filenames = {
%     'sample_1.tif'
%     'sample_2.tif'
% };

%% Scale calibration

img = imread(fullfile(img_folder,filenames{1}));

if size(img,3) == 3
    img_gray = rgb2gray(img);
```

```
else
    img_gray = img;
end

figure;
imshow(img_gray,[]);
title('Select the beginning and end of the scale bar');

[x,y] = ginput(2);
close;

real_length_um = input('Enter the real scale bar length [um]: ');

scale_bar_pixels = sqrt((x(2)-x(1))^2 + (y(2)-y(1))^2);
um_per_pixel = real_length_um / scale_bar_pixels;

fprintf('\nCalculated scale: %.4f um/pixel\n',um_per_pixel);

%% Threshold parameter

scale_factor = input('Enter threshold scaling factor: ');

%% Initialize results

nImages = numel(filenames);

mean_diameters = zeros(1,nImages);
std_diameters  = zeros(1,nImages);
all_diameters  = cell(1,nImages);

%% Image processing loop

for f = 1:nImages

    fprintf('\nProcessing image %d of %d\n',f,nImages);

    img = imread(fullfile(img_folder,filenames{f}));

    if size(img,3) == 3
        img_gray = rgb2gray(img);
    else
        img_gray = img;
    end

    % Crop image to remove SEM scale bar and metadata
    [rows,~] = size(img_gray);
    img_crop = img_gray(1:round(0.85*rows),:);

    % Contrast enhancement
    img_eq = adapthisteq(img_crop);

    % Thresholding
    level = graythresh(img_eq) * scale_factor;
    bw = imbinarize(img_eq,level);

    % Ensure that fibers correspond to foreground pixels
    if mean(bw(:)) > 0.5
        bw = ~bw;
    end

    %% Fiber diameter analysis

    skeleton = bwmorph(bw,'skel',Inf);
```

```matlab

    dist_map = bwdist(~bw);

    fiber_radius_map = dist_map .* skeleton;

    radii_px = nonzeros(fiber_radius_map);

    diameters_um = 2 * radii_px * um_per_pixel;

    mean_diameters(f) = mean(diameters_um,'omitnan');
    std_diameters(f)  = std(diameters_um,'omitnan');

    all_diameters{f} = diameters_um;

    %% Optional visualization

    figure;
    imshow(bw,[]);
    title(sprintf('Binarized image %d',f));

end

%% Final statistics

group_mean = mean(mean_diameters,'omitnan');
group_std  = std(mean_diameters,'omitnan');

fprintf('\nFinal results\n');
fprintf('Mean fiber diameter: %.2f um\n',group_mean);
fprintf('SD fiber diameter: %.2f um\n',group_std);

%% Optional bar plot

figure('Color','w');
hold on;

b = bar(mean_diameters, ...
    'FaceColor',[0.20 0.45 0.70], ...
    'EdgeColor','none');

errorbar(1:nImages,mean_diameters,std_diameters, ...
    'k', ...
    'LineStyle','none', ...
    'CapSize',10, ...
    'LineWidth',1.2);

set(gca, ...
    'XTick',1:nImages, ...
    'XTickLabel',compose('Image %d',1:nImages), ...
    'FontSize',12, ...
    'LineWidth',1.1);

ylabel('Fiber diameter [um]');
xlabel('SEM images');

box off;
grid off;
```

Listing S5: MATLAB script used for fiber diameter analysis of electrospun scaffolds from SEM images.
